# The Role of Calmodulin in Tumor Cell Migration, Invasiveness, and Metastasis

**DOI:** 10.3390/ijms21030765

**Published:** 2020-01-24

**Authors:** Antonio Villalobo, Martin W. Berchtold

**Affiliations:** 1 Cancer and Human Molecular Genetics Area—Oto-Neurosurgery Research Group, University Hospital La Paz Research Institute (IdiPAZ), Paseo de la Castellana 261, E-28046 Madrid, Spain; 2 Department of Biology, University of Copenhagen, 13 Universitetsparken, DK-2100 Copenhagen, Denmark

**Keywords:** calcium signaling, calmodulin, calmodulin antagonists, cell migration, tumor cell invasiveness, metastasis

## Abstract

Calmodulin (CaM) is the principal Ca^2+^ sensor protein in all eukaryotic cells, that upon binding to target proteins transduces signals encoded by global or subcellular-specific changes of Ca^2+^ concentration within the cell. The Ca^2+^/CaM complex as well as Ca^2+^-free CaM modulate the activity of a vast number of enzymes, channels, signaling, adaptor and structural proteins, and hence the functionality of implicated signaling pathways, which control multiple cellular functions. A basic and important cellular function controlled by CaM in various ways is cell motility. Here we discuss the role of CaM-dependent systems involved in cell migration, tumor cell invasiveness, and metastasis development. Emphasis is given to phosphorylation/dephosphorylation events catalyzed by myosin light-chain kinase, CaM-dependent kinase-II, as well as other CaM-dependent kinases, and the CaM-dependent phosphatase calcineurin. In addition, the role of the CaM-regulated small GTPases Rac1 and Cdc42 (cell division cycle protein 42) as well as CaM-binding adaptor/scaffold proteins such as Grb7 (growth factor receptor bound protein 7), IQGAP (IQ motif containing GTPase activating protein) and AKAP12 (A kinase anchoring protein 12) will be reviewed. CaM-regulated mechanisms in cancer cells responsible for their greater migratory capacity compared to non-malignant cells, invasion of adjacent normal tissues and their systemic dissemination will be discussed, including closely linked processes such as the epithelial–mesenchymal transition and the activation of metalloproteases. This review covers as well the role of CaM in establishing metastatic foci in distant organs. Finally, the use of CaM antagonists and other blocking techniques to downregulate CaM-dependent systems aimed at preventing cancer cell invasiveness and metastasis development will be outlined.

## 1. Introduction

**Figure 1:**
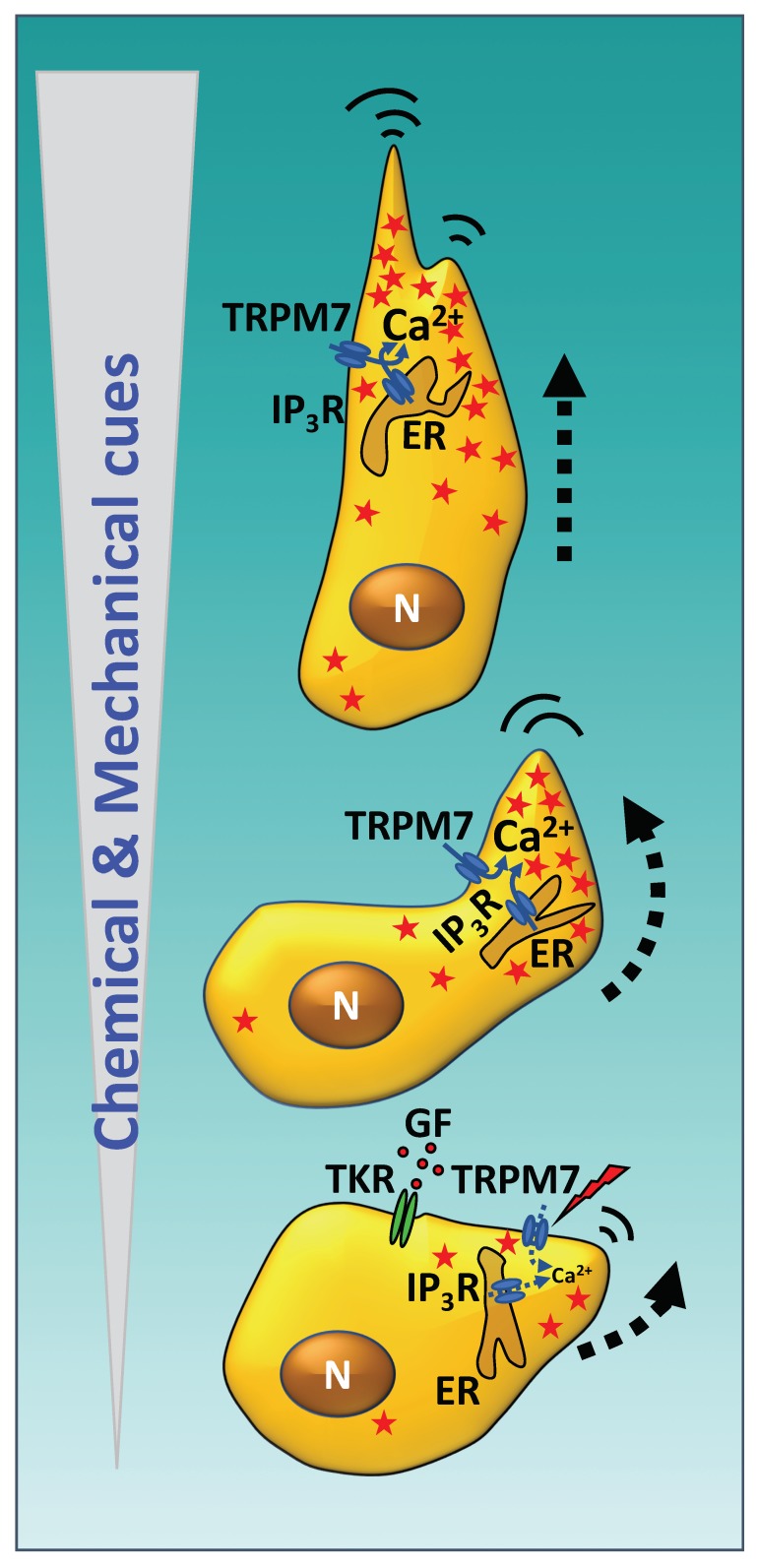
**Directional steering of cell movement by Ca^2+^ flickering following chemical and mechanical cues.** Resting cells sensing an increasing chemical gradient of growth factors (e.g., VEGF, EGF—*red dots*) activate their respective tyrosine kinase receptors (TKR) and downstream signaling which opens the IP_3_R channel located in the endoplasmic reticulum (ER). Likewise, mechanical cues (*lightning symbol*), such as fluid flow, initiate the translocation of TRPM7 to the plasma membrane. The synchronous opening of both Ca^2+^ channels located in the ER and plasma membrane at or near the front edge of the cell generates brief Ca^2+^ flickers (*red stars*) in the cytosolic region where cell movement will be initiated. Upon an increment in the intensity of the chemical and mechanical cues, the number of flickering events dramatically increases in the leading front of the cell, which turns in the appropriate direction, initiating migration following the increasing chemical gradient. Notice the higher number of flickering events in the front part of the cytosol as compared to the rear end. EGF, epidermal growth factor; GF, growth factor; IP_3_R, inositol 3-phosphate receptor; N, nucleus; TRPM7, transient receptor potential melastatin channel 7; VEGF, vascular endothelial growth factor. See text and references [2,3,4] for more details.

Calcium is a widespread second messenger that controls a variety of mechanisms required for cell motility. A pioneering discovery was the demonstration that migrating eosinophils during chemotaxis develop a Ca^2+^ gradient in the cytosol between the rear trailing end and the leading progressing front of the cell, in which the concentration was lower in the latter than in the former [1]. One reason for this is the enrichment of plasma membrane Ca^2+^-ATPase in the plasmalemma of the advancing cellular front, which pumps Ca^2+^ out of the cell causing the lower concentration of free Ca^2+^ in this region of the cytosol (reviewed in [2]). As pointed out by Wei et al. [3], this asymmetric Ca^2+^ distribution appears at first sight paradoxical, as the leading front is where cells accumulate systems necessary for cell migration requiring Ca^2+^ for their functionality, such as those controlling cytoskeleton remodeling or the assembly and dynamics of focal adhesions. This apparent contradiction was dispelled by the discovery that synergistic opening of the mechanosensitive Ca^2+^ channel TRPM7 (transient receptor potential melastatin channel 7) and endoplasmic reticulum-located IP_3_R (inositol 3-phosphate receptor), generate small Ca^2+^ flickers which are short in duration (ranging from 10 ms to 2 s), predominantly occurring at the advancing front edge of the cell, which activate the Ca^2+^-dependent systems, including calmodulin (CaM) and CaM-dependent systems, required for cell migration [3] (reviewed in [4]). Furthermore, these authors demonstrated that there was an increment in the frequency of Ca^2+^ flickering in the cytosol region coinciding with the direction where the cell moves following mechanical and chemical cues. This ground-breaking discovery demonstrated that the steering of the cell toward appropriated chemotactic paths is regulated by Ca^2+^ signaling [3] (reviewed in [2,4]). A recent detailed review by Dasgupta and McCollum [5] describes in detail how cells transduce mechanical stimuli derived from substrate stiffness, F/G-actin levels or actomyosin-transmitted tension sensed by focal adhesions, adherent junctions and other factors by the Hippo-signaling pathway kinases LATS1/2 (large tumor suppressor kinases 1/2) as well as by FAK (focal adhesion kinase) and c-Src. These pathways regulate the homologous transcriptional co-activators YAP (Yes-activated protein) and TAZ (transcriptional coactivator with PDZ-binding motif) in a negative and positive way, respectively, and thereby control transcriptional programs required for cell motility and other cellular responses. Figure 1 shows in a schematic manner how a cell subjected to a gradient of increased chemical and mechanical cues induces an increment of cytosolic Ca^2+^ flickering in the lead moving front that steers the cell toward the direction of higher concentration/intensity by the synchronous opening of TRPM7 and IP_3_R channels. Of note, the translocation of TRPM7 from intracellular tubulovesicular structures to the plasma membrane has been shown to occur very rapidly in less than two minutes when cells were exposed to a mechanical stimulus such as a laminal fluid flow [6]. For reviews on TRP channels including TRPM7 see [7], and for the molecular basis of mechano-transduction see [8,9].

The role of Ca^2+^ in tumor cell migration and the development of metastasis has been recently reviewed [2], pointing to the relevance and some anomalies of certain Ca^2+^ transport systems. In this regard, the endoplasmic reticulum Ca^2+^ sensor STIM1, regulator of Orai channels, was first identified as a promoter of lung metastasis [10]. In fact, Orai1 is overexpressed in some tumors [11], and downregulation of both STIM1 and Orai1 by siRNA or directly blocking store-operated Ca^2+^ entry with the drug SKF96365 (1-[2-(4-methoxyphenyl)-2-[3-(4-methoxyphenyl)propoxy]ethyl]imidazole) inhibited tumor cell migration and metastasis development [12,13]. Furthermore, the non-selective Ca^2+^ channel TRPM7 is overexpressed in human breast tumor cells, and this channel was required for metastasis development in an experimental animal model [14]. These examples underscore the relevance of Ca^2+^ mobilization for tumor cell invasiveness and metastasis. However, intracellular Ca^2+^ overload inhibited the migration of human melanoma cells via a calcineurin-mediated mechanism, a process that was restored by decreasing cytosolic Ca^2+^ [15].

Frequency- and intensity-modulated fluctuations in the cytosolic, nuclear, and other organelles free Ca^2+^ concentration are transduced into signals able to control multiple molecular systems and cellular functions by the actions of a number of regulatory Ca^2+^-binding proteins. Among these, the four EF-hand-containing Ca^2+^-receptor protein CaM has been shown to be a major ruler in all eukaryote cells upon binding to target proteins.

## 2. Calmodulin

One advantage for the evolutionary selection of CaM for exerting multiple regulatory roles in eukaryote cells is due to three main factors. First is its versatility in recognizing a diversity of protein motifs in hundreds of target proteins, encompassing the recognition of structural features such as basic amphiphilic α-helices [16]. In addition to general recognition structures, CaM is able to recognize specific sequence features, such as pairs of conserved bulky hydrophobic amino acid residues at constant distances between them, forming three motif-families denoted 1-10, 1-14 and 1-16, which include several subclasses (reviewed in [17,18], data available at http://calcium.uhnres.utoronto.ca/ctdb). Further, CaM specifically interacts with the so-called IQ motifs with the consensus sequence (I,L,V)QxxxRxxxx(R,K) or IQ-like motifs with the consensus sequence [(FILV)Qxxx(RK)Gxxxxxxxx], where x represents any amino acid (reviewed in [19,20]). Secondly, the high flexibility of its central linker plays an important role for the versatility of CaM in target binding. This allows the reorientation of its N- and C-terminal Ca^2+^-binding lobes at different angles, facilitating their alternative independent interaction either with two different sites of the same target protein, forming in this way new structural features that have functional consequences, or working as an adaptor protein linking two identical or distinct proteins, allowing the formation of homo- or hetero-dimers (reviewed in [21]) This regulation mostly occurs when CaM is Ca^2+^-bound, however to a lesser extent also in its Ca^2+^-free form. Thirdly, of major importance is the unusual content of methionine in CaM. Of the 148 amino acids residues 9 are methionine. It has been shown that 50% of the hydrophobic patch areas used for target interaction are occupied by methionine in the Ca^2+^-bound form of CaM, which exhibits unusual flexibilities in protein interaction, thus methionine exposure in Ca^2+^/CaM is critical for the recognition of target proteins [22,23].

CaM regulates the functionality of many kinases, phosphatases, as well as many other enzymes, channels, transport systems, transcription factors, and structural, signaling, and adaptor/scaffold proteins, among others (reviewed in [19,21,24,25,26,27]). Moreover, phosphorylation of CaM by both Ser/Thr- and Tyr-kinases leads to altered regulation of various target proteins as compared to its non-phosphorylated form, suggesting that phosphorylation exerts a fine-tuning in CaM functionality (reviewed in [28,29]). In this review, we discuss the implication of CaM in the migration of tumor cells, as well as its role in the control of molecular processes responsible for their transformation into highly migrating invasive cells, key factors playing crucial roles in the initial steps of the development of metastasis. To understand the anomalous behavior of tumor cells in this respect, we also summarize in this article basic mechanisms involved in the migration of non-tumor cells, in order to provide a general view of the molecular mechanisms implicated. Finally, we highlight the attempts to inhibit tumor cell migration and invasiveness by blocking CaM-dependent systems using CaM antagonists and other techniques. These studies could be the basis for the development of potential therapeutic strategies to delay or prevent metastasis development.

## 3. Calmodulin and Cell Migration

**Figure 2:**
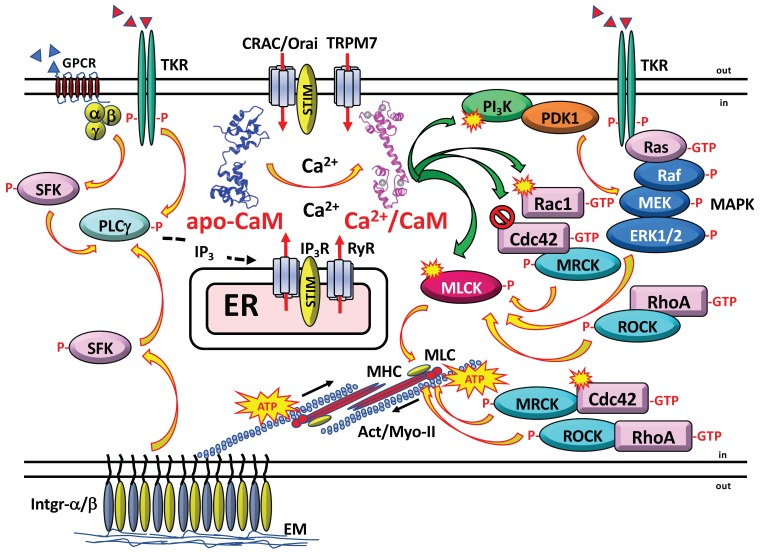
**The Ca^2+^ signal and calmodulin (CaM)-mediated regulation of actomyosin contraction.** Activation of GPCR and TKR by their respective ligands (*blue and red triangles*), and Intgr-α/β engagement to extracellular matrix proteins induce a Ca^2+^ transient. This is due to Ca^2+^ release from internal stores such as IP_3_R and RyR, and Ca^2+^ entry via plasma membrane-located Ca^2+^ channels such as Orai1/CRAC, when Ca^2+^ in the endoplasmic reticulum lumen drops and is sensed by STIM, or TRPM7 among others. The activation of PLCγ by TKR and SFK, as intermediates of GPCR and Intgr-α/β, are also depicted. The cytosolic Ca^2+^ rise increases the transitions of apo-CaM to Ca^2+^/CaM which activates MLCK, PI_3_K, the small G-protein Rac1 while inhibiting Cdc42. MLCK and downstream Rac1/Cdc42-activated MRCK and RhoA-activated ROCK phosphorylate myosin-II within the Act/Myo-II complex generating sliding movements, which leads to contraction of F-actin fibers and cell motility. Activation of the MAPK pathway by TRK, activation of MEK by PI_3_K/PDK1, and phosphorylation of MLCK by MRCK, ROCK and ERK1/2 are also illustrated. The active forms of small GTPases and kinases are indicated with the symbols *-GTP* and *-P*, respectively. The structures of *Xenopus laevis* apo-CaM (ID: 1DMO) [39] and human Ca^2+^/CaM (ID: 1CLL) [40] were obtained from the Protein Data Bank. α,β,γ, trimeric G protein α,β,γ-subunits; Act/Myo-II, actomyosin; apo-CaM, apo-calmodulin; Ca^2+^/CaM, Ca^2+^/calmodulin; CRAC/Orai, Ca^2+^ release-activated channel; EM, extracellular matrix; ER, endoplasmic reticulum; ERK1/2, extracellular regulated kinases-1/2; GPCR, G protein-coupled receptor; Intgr-α/β, integrins-α/β; IP_3_, inositol 3-phosphate; IP_3_R inositol 3-phosphate receptor; MAPK, mitogen-activated protein kinase; MEK, mitogen-activated ERK-1/2 kinase; MHC, myosin heavy-chain; MLC, myosin light-chain; MLCK, myosin light-chain kinase; MRCK, myotonic dystrophy kinase-related Cdc42-binding kinase; PDK1, phosphoinositide-dependent kinase-1; PI_3_K, phosphatidyl-inositol 3-kinase; PLCγ, phospholipase Cγ; ROCK, Rho-kinase; RyR, ryanodine receptor; SFK, Src-family kinase; STIM, stromal interacting molecule; TKR, tyrosine kinase receptor; TRPM7, transient receptors potential melastatin channel 7. See text and reference [41] for more details.

Cells use focal adhesions, which are points of attachments to the extracellular matrix, for cell migration. These dynamic structures located at the plasma membrane connect the actin cytoskeleton to the extracellular matrix via clusters of integrins and a series of actin-binding proteins and associated signaling proteins, some of which are regulated by CaM as described below. Focal adhesions exert signaling and mechano-transducing functions by transmitting the sliding movements of bundles of actomyosin stress fibers inducing motility and cell propulsion (reviewed in [9,30,31,32,33]). Podosomes are other structures involved in cell migration. These are complex and highly dynamic integrin-mediated cell adhesion circular structures in the plasma membrane connected to the actin cytoskeleton with mechano-transducer function that locally dissolve the extracellular matrix in a restricted manner by associated metalloproteases (reviewed in [34,35,36,37]). Ca^2+^ signaling plays an important role in the assembly of podosomes. For example, podosomes are required for the migration of microglia cells across the brain parenchyma, as these structures contain, among other components, CaM, Orai1/CRAC (calcium release-activated) channels, Ca^2+^-activated small-conductance K^+^ channels SK3 and other CaM-regulated proteins [38]. Cancer cells utilize their Ca^2+^ and CaM-dependent signaling system to enhance their migration rate and invasion efficiency. Figure 2 shows a general overview of CaM activation by a Ca^2+^ transient and the implication of Ca^2+^/CaM in different systems involving actomyosin contraction leading to cell migration as discussed below.

CaM in human and other mammals is encoded by three non-allelic genes denoted *Calm1,2,3* even though the three distinct CaM transcripts yield an identical CaM protein [42]. However, although a given cell could express the three genes, not necessarily all have the same functional role, as the three transcripts could be differentially processed by post-transcriptional regulation or subcellular distribution (reviewed in [43]). Highlighting this point was the demonstration that only *Calm1* was necessary for the migration of mouse precerebellar neurons (PCNs) as determined in vivo. Single migrating PCNs express the three CaM genes, and their relative expression is *Calm1* > *Calm2* > *Calm3*, whereas the expression of *Calm2* and *Calm3* is 66% and 19%, respectively, of the level of *Calm1* mRNA. Nevertheless, CaM derived from the *Calm2* and *Calm3* genes combined did not functionally replace *Calm1* expression, possibly because their mRNAs are less efficiently translated. This was demonstrated by knocking down *Calm1* with shRNA, resulting in limited radial and tangential migration of the cells, which failed to reach their final destination during development, while knocking down *Calm2* and *Calm3* did not have any deleterious effect [44].

The implication of CaM in non-tumor cell migration has been tested using a great variety of CaM antagonists (see Table 1). For example, *N*-(6-aminohexyl)-5-chloro-1-naphthalenesulfonamide (W-7), calmidazolium (R24571), trifluoperazine (TFP), chlorpromazine, and other derived compounds have been shown to inhibit cell spreading and the migration of a variety of cells tested using different methods [45,46,47,48,49,50,51]. These and other less known CaM antagonists, such as flunarizine [52], exert similar effect in tumor cells [53,54] (see Table 2). These compounds inhibit the adhesion and migratory capacity of endothelial vascular cells induced by hypoxia, a CaM-dependent process required for angiogenesis [55]. Nevertheless, it was reported that neither TFP nor W-7 at relatively high concentrations up to 10 μM significantly inhibited the migration of fetal endothelial aortic cells [56], and do not have significant effect (W-7) or only a somewhat small effect (TFP) inhibiting the transmigration of monocytes [57]. In addition, TFP and calmidazolium failed to significantly affect Madin–Darvy canine kidneys cell detachment from each other induced by the scatter factor [58], suggesting lack of an inhibitory effect on cell motility. The reason for the discrepancies in the inhibitory potency among different CaM antagonists or among different cell types is unknown. Moreover, TFP and ophiobolin A after inhibiting CaM, enhance Ca^2+^ leakage from the endoplasmic reticulum. This mimics the phenotype observed after downregulating the Sec62 subunit from the Sec62/63 dimer with siRNA, as Sec62 senses and prevents passive Ca^2+^ leak via the heterotrimeric Sec61 channel complex. Binding of Ca^2+^/CaM seals the channel resulting in decreased cell migration [59]. A didactic review on the role of the Sec61/62/63 system in protein translocation of nascent and precursor polypeptides into the endoplasmic reticulum (ER), passive Ca^2+^ leakage from the ER, and its implication in human diseases including cancer has recently been published [60]. This demonstrates that data obtained from the use of CaM antagonists in intact cells could lead to misinterpretation of observed changes in cellular functions, as these compounds may give rise to side effects derived from cytosolic Ca^2+^ overload due to the described opening of the Sec61 Ca^2+^-leak channel. 

Inflicting small scratches in corneal tissues results in the reepithelization of the wound. In rat cornea it was found that the CaM-antagonists trifluoperazine and W-7 inhibit the repair of the wounds, but not in rabbit cornea [88]. This disparity was confirmed by measuring the migration of epithelial corneal cells of both species in cell culture [64]. It is important to mention, however, that the tissular assay is a poor measure for cell migration, as proliferation also participates in the reepithelization of the wound. This is confirmed using artificial wound healing assays in a monolayer of rabbit gastric mucosal cells, where cell migration occurs during the early phase of the wound repair followed by cell proliferation [66], processes that were inhibited by either W-7 or calmidazolium [61,66]. Indeed, the inhibitory action of CaM antagonists on cell proliferation is well established (see for review [27]).

The inhibition of the migratory capacity of T lymphocytes through a fibroblast monolayer has been tested using different phenothiazines and other compounds that are known to antagonize CaM [73]. Trifluoperazine, chlorpromazine, and to a lesser extent W-7, inhibited T-cell migration through a porous membrane, while diphenylbutylpiperidine lacked an inhibitory effect. Surprisingly, the authors of this study observed that trifluoperazine and chlorpromazine inhibited the invasive capacity of the T cells by a CaM-independent mechanism, while diphenylbutylpiperidine, a more potent CaM antagonist but weaker migratory inhibitor, inhibited the invasion of the T cells by a CaM and CaM-dependent protein kinase-II (CaMK-II)-dependent pathway [73]. The transmigration of Vδ2^+^TCRγδ^+^ T cells also utilizes signaling by CaMK-II after engagement of natural killer receptor protein 1a (NKRP1a), as demonstrated utilizing specific inhibitors of this kinase, while Vδ1^+^TCRγδ^+^ T cells lacking NKRP1a use instead PECAM1 (platelet endothelial cell adhesion molecule 1) and the phosphatidylinositol 3-kinase (PI_3_K)/Akt pathway for transmigration [89]. The migration of neutrophils induced by the chemo-attractants IL-8 or *N*-formyl-methionyl-leucyl-phenylalanine was suppressed by W-7, suggesting a role of Ca^2+^/CaM in this process [67]. Praeruptorin A is a phytocompound isolated from an herbaceous plant that inhibits receptor activator of NFκB ligand (RANKL)-induced migration of preosteoclasts, and based on the fact that analogues of this molecule have been shown to bind CaM, it was proposed that its action was due to the inhibition of CaM and hence the downstream CaMK-IV/CREB and calcineurin/NFATc (nuclear factor of activated T cells) signaling pathways [90]. Calcineurin in fact plays an important role in cell migration. Thus, inhibition of calcineurin with a specific peptide, or the immunosuppressive drug FK506 (tacrolimus) bound to its target immunophilin, inhibits the migration of neutrophils on a vitronectin matrix [91]. The transmigration of monocytes from the blood stream toward inflammation sites in damaged tissues requires the participation of L-selectin. This adhesion molecule sheds its ectodomain after proteolytic cleavage by metalloproteases during the transmigration process. The binding of CaM to L-selectin prevents its shedding, while its phosphorylation, most probably by PKC, liberates CaM from L-selectin allowing its shedding, a process that exclusively occurs in L-selectin molecules located in the protruding pseudopods at the front leading edge of the migrating monocyte, but not in L-selectin molecules located in other places [92].

Of note, human CaM-like protein (CLP) is specifically expressed in epithelial cells, has four EF-hand Ca^2+^ binding sites with eight-fold lower affinity for this cation compared to CaM, shares with the latter the capacity to regulate, however with different efficiency, identical target proteins, and therefore both proteins may have some overlapping and/or competitive functions [93,94,95]. CLP also participates in cell motility, and in this context, it has been shown in HeLa carcinoma cells and human keratinocytes transfected with CLP that the expression of the unconventional myosin-10 is upregulated. Myosin-10 is associated with actin filaments in filopodia and enhances cell migration in wound healing assays when upregulated [96,97]. The authors of these studies also suggested that CLP participates in the reepithelization of wounds in vivo. Of interest for future studies would be to determine whether CLP competes with CaM modulating the activity of CaM-binding proteins implicated in cell migration.

An interesting cooperation between the Na^+^/K^+^-ATPase and the Na^+^/Ca^2+^-exchanger in cell migration has been unveiled [98]. Stoichiometric efflux of 1 Ca^2+^ ion in exchange for the uptake of 3 Na^+^ ions is driven by the high Na^+^ concentration in the extracellular medium and the membrane potential (negative inside) generated by the Na^+^/K^+^-ATPase. This system helps to maintain the cytosolic Ca^2+^ concentration ([Ca^2+^]_cyt_) low in basal conditions. Binding of the regulatory β-subunit of the ATPase to the Na^+^/Ca^2+^-exchanger inhibits its activity, which increases the [Ca^2+^]_cyt_ that in turn induces cell migration. This is due to the Ca^2+^/CaM-mediated activation of PI_3_K that activates the mitogen-activated protein kinase (MAPK) pathway resulting in the phosphorylation of myosin light-chain via myosin light-chain kinase (MLCK) and Rho-kinase (ROCK) [98] (see Figure 2).

### 3.1. CaM and Cytoskeleton Dynamics

The regulation of cytoskeleton dynamics by Ca^2+^/CaM, either activating MLCK, which phosphorylates the light-chain of myosin, or negatively acting on microtubules has long been recognized as reviewed by Means and collaborators decades ago using Sertoli cells as an experimental model [99]. MLCK is a CaM-dependent kinase that plays a fundamental role regulating cell migration and function as a key player in smooth, skeletal, and cardiac muscle contraction (reviewed in [41,100,101]). Inhibition of MLCK and CaM by specific inhibitors (ML-7 and W-7, respectively), prevents the migration of myofibroblasts induced by cardiotrophin-1 participating in the repair of scars after infarction [68]. Dupuytren’s disease is an idiopathic fibroproliferative condition characterized by the formation of nodular lesions in the palmar fascia where the accumulation of fibroblasts and highly contractile myofibroblasts severely alters the function of the hands (reviewed in [102]). The CaM antagonist fluphenazine inhibited the migration of fibroblasts isolated from the collagen lattice of pathological lesions of patients with Dupuytren’s disease, and this was attributed to MLCK inhibition [62].

The MAPK pathway participates in MLCK function, as its phosphorylation by extracellular-regulated kinases 1/2 (ERK1/2) enhances its sensitivity to Ca^2+^/CaM and the phosphorylation of myosin light-chain, which in turn activates the actin/myosin motor required for cell migration [103]. The crystal structure of myosin-1c in complex with Ca^2+^/CaM reveals two points of interaction implicating the motor domain and the first IQ motif located in the neck region of myosin [104]. The inhibition of MLCK reduces cell migration, but this depends on the migratory rate of the cells. Fast migrating leukocytes depend on non-muscle myosin-II activity, and hence MLCK, while slow spontaneously moving tumor cells only use this system when the migration rate is speeded up upon stimulation with chemotactic factors [105]. The Ca^2+^ signal responsible for activation of the CaM/MLCK system is generated by the opening of L-type voltage-dependent Ca^2+^ channels leading to the contraction and detachment of the trailing-end of migrating fibroblasts [106]. Moreover, MLCK also controls cell spreading by promoting the dynamic assembly of zyxin-containing focal adhesions at their advancing leading edge [107].

Caldesmon is a cytoskeletal protein present in smooth muscle cells and non-muscle cells and is regulated by direct binding of CaM in addition to phosphorylation. Caldesmon stabilizes the actin filaments, regulates the interaction between actin and myosin, and plays, among other functions, a fundamental role in cell migration by reorganizing the actin cytoskeleton (reviewed in [108,109]). Transfection of CHO cells with a mutant caldesmon with two tryptophan residues substituted by alanine at either of its two CaM-binding sites disrupted the assembly of actin-stress fibers and focal adhesions, negatively affecting cell migration [110]. This underscores the function of Ca^2+^/CaM in transmitting signals during cell migration by regulating caldesmon functionality. Overexpression of caldesmon in vascular endothelial cells induced the assembly of a thick peri-cellular subcortical rim of actin fibers concomitant with decreased migration [111]. Filamin is an actin filament crosslinking protein that is phosphorylated by CaMK-II. A cell-permeable peptide corresponding to the CaMK-II phosphorylation site decreased the migration of endothelial cells, stressing the importance of F-actin branching in cell motility [112].

Vimentin is a major intermediate filaments protein with multi-functional roles in the cell, participating in the directional migration of multiple cell types through modulation of microtubules and actomyosin dynamics (reviewed in [113,114]). The dynamic phosphorylation/dephosphorylation of vimentin is a key factor in its functionality, and Ca^2+^/CaM-dependent phosphorylation of this protein in Sertoli cells, as well as its CaMK-II-dependent phosphorylation in T cells has been demonstrated, suggesting an important role in the dynamics of intermediate filaments [115,116]. Furthermore, CaMK-II also phosphorylates MAP2 (microtubule-associated protein 2) [117], a major microtubule protein that in addition to its role in dendritic outgrowth, also synergistically participates together with MAP1B in cell migration. This was demonstrated using double knockout MAP2^−/−^ and MAP1B^−/−^ mice showing retarded neuronal migration and impaired brain cytoarchitecture [118]. In this context, it is important to mention that the thyroid hormone T_3_ regulates CaMK-IV through a thyroid hormone-responsive element in its promoter [119]. Microarray analysis of gene expression in the cerebral cortex of hypothyroid and normal rat fetuses showed differential expression of genes related to cytoskeletal reorganization, cell migration, and dendritic projections, among other processes. At least 20% of these genes were related to CaMK-IV signaling pathways, pointing to the importance of the CaMK-IV pathway in causing altered brain cytoarchitecture in hypothyroidism, which contributes to mental disability [120]. The Ca^2+^/CaM-binding protein denoted p35 also plays an important role in microtubule polymerization and dynamics affecting neuronal cell migration, in addition to its role in the extension of neurites and axonal growth. The CaM-binding site of p35 overlaps with the site of interaction with the microtubules, explaining the role of Ca^2+^/CaM inhibiting the assembly of microtubules, a process that is attained by the phosphorylation of p35 at Thr138 by Cdk5 [121].

### 3.2. CaM-Dependent Phosphorylation

In addition to the essential role of MLCK-mediated phosphorylation as discussed in the previous section, other CaM-dependent kinases participate in cytoskeleton dynamics important for cell migration. In this section we will discuss phosphorylation events catalyzed by CaMK-II, CaMK-D1, CaM-dependent kinase kinase-β (CaMKKβ)/CaMK-IV and CaMK-III.

CaMK-II is accepted as a prominent player in cell migration by acting at different levels. However, some conflicting results exist concerning its specific role in this process. For excellent reviews discussing the role of CaMK-II in vascular smooth muscle cells (VSMCs) motility, and other processes, in response to Ca^2+^ mobilization by different effectors see [122,123]. The migration of VSMCs in response to platelet-derived growth factor (PDGF), or upon artificially increasing the intracellular concentration of Ca^2+^ induced by ionomycin treatment, requires the intervention of CaMK-II as demonstrated using kinase inhibitors or the CaM antagonist W-7 [69,124]. In this system, CaMK-II is working downstream of the PDGF-mediated activation of the MAPK pathway, although MAPK activation is also able to induce cell migration by an alternative route independent of CaMK-II, suggesting a dual path to achieve PDGF-induced chemotaxis [125]. The knockdown of histone deacetylase 4 with siRNA inhibited PDGF-stimulated migration of VSMCs preventing lamellipodia formation. The implication of this deacetylase in migration and proliferation of these cells during neointimal hyperplasia involves a pathway that is dependent of CaMK-II [69].

Activated CaMK-IIδ2 is located in the front leading edge of VSMC during polarized cell migration and seems to be involved in activation of Rac1 and the MAPK pathway as demonstrated by knocking down CaMK-IIδ with siRNA and transfection with a dominant negative kinase-death mutant version of the kinase [126]. CaMK-IIδ also phosphorylates CREB at Ser142 inhibiting its transcriptional activity by preventing DNA binding [127]. This provides an additional evidence of the dual regulation of CREB by negative (Ser142) and positive (Ser133) phosphorylation events [128]. These results underscore that regulation of cell migration by CaMK-IIδ/CREB signaling may be subjected to a fine reversible tuning. A direct functional interaction between CaMK-IIδ_2_ and the downstream Src-family kinase Fyn has been demonstrated. This is followed by the activation of Fyn mediated by the previous dephosphorylation of its regulatory Tyr527 residue, and hence the coordinated regulation of VSMC migration by both kinases [129].

A crosstalk between different integrin isoforms has been recognized. Integrin-α_5_β_1_ engagement to fibronectin activated CaMK-II, inducing cell migration. However, simultaneous ligation of transfected integrin-α_v_β_3_ inhibited integrin-α_5_β_1_-mediated cell migration, a process that can be reversed by expressing a constitutively active CaMK-II mutant [130]. Another point of action of CaMK-II relates to the dynamic control of focal adhesions as demonstrated in other cell types. This was done by phosphorylating the negative regulator of integrin-β_1_ denoted ICAP-1α (integrin cytoplasmic domain-associated protein 1α) at Thr38, which upon phosphorylation exposed the integrin-binding site and maintained integrin-β_1_ in an inactive conformation unable to recruit talin, and hence the attachment of actin stress fibers required for cell migration [131].

The role of Ca^2+^ in the scattering of epithelial cells after EMT during development involves the activation of TRP channels (reviewed in [132]). In its inactive form CaMK-II favors actin bundling, while activation of the c-Met tyrosine kinase receptor by scattering-factor/hepatocyte growth factor (HGF) induces Ca^2+^ entry via TRP channels. This is followed by the activation of CaM/CaMK-II, which in turn phosphorylates filamin and spinophilin dismantling the actin bundles, as the former proteins cross-link the individual actin filaments. Moreover, CaMK-II has a dual role in cell contractility by phosphorylating MLCK, which inactivates the regulatory light-chain of myosin and decreases cell contractility, and by activating RhoA, inducing cell contractility via ROCK, which activates the myosin light-chain (reviewed in [132]).

The CaMK-II inhibitor KN-93 (*N*-[2-[*N*-(4-chlorocinnamyl)-*N*-methylaminomethyl]phenyl]-*N*-(2-hydroxyethyl)-4-methoxybenzenesulfonamide) exerts an inhibitory effect on vascular endothelial cell migration during revascularization after heart ischemic conditions, underscoring the fundamental role that this kinase plays in this process [133]. The required formation of the Ca^2+^/CaM complex to induce migration of endothelial cells is dependent on the entry of extracellular Ca^2+^ in the cell or its release from intracellular stores during hypoxia via a variety of Ca^2+^ channels (reviewed in [134]). The migration of retinal endothelial cells during angiogenesis induced by vascular endothelial growth factor (VEGF) stimulation is linked to the ensuing transient Ca^2+^ increase and the subsequent activation of CaMK-II [135]. Moreover, CaMK-II is very important for the migration of neural cells during development. In this context and of medical interest, it has been demonstrated that mutations affecting the autophosphorylation of CaMK-IIa and CaMK-IIb impair neural cell migration, suggesting that these mutations affect normal neurodevelopment and are the culprits for the intellectual disability observed in some patients [136].

In human fibroblasts and fibrosarcoma cells, CaMK-II was shown to phosphorylate the cell adhesion receptor CD44 (cluster of differentiation 44) at Ser325 in vitro and in vivo [137]. This receptor binds to its ligand glycosaminoglycan hyaluronan, which is required for cell migration through the extracellular matrix. However, this does not explain how CD44 phosphorylation by CaMK-II regulates cell migration. On the other hand, in head and neck carcinoma cells it was established that activation of CD44 in complex with the EGFR (epidermal growth factor receptor) signals the release of Ca^2+^ from the endoplasmic reticulum, leading to CaMK-II activation. In turn, CaMK-II was shown to phosphorylate filamin reducing its interaction with actin filaments and therefore inducing cell migration [138]. The mechanistic role of CaMK-II in TGFα-induced CD44 expression after activating the transcriptional complex AP-1 was established in monocytes, although an additional pathway, independent of CaMK-II, induced by polysaccharides with the participation of JNK (c-Jun N-terminal kinase) and the transcription factor EGR-1 (early growth response protein-1) also controls CD44 expression [139].

CaMK-D1 is another CaM-dependent kinase participating in cell motility, particularly in the migration and invasion of trophoblasts during embryo implantation in the endometrium [140] and in the migration of cells during VEGF-induced angiogenesis (reviewed in [141]). An additional CaM-dependent kinase with restricted expression in CD34^+^-derived neutrophils, eosinophils, and circulating human granulocytes is CaMK-I-like (CKLiK). Among other functions this kinase controls the activation of integrin-β_2_ and hence the attachment and migration of these leukocytes. This was demonstrated using a cell-penetrating peptide comprising the CaM-binding and autoinhibitory domains of CKLiK, similar to those in CaMK-I [142], which inhibits its activity and chemoattractant-induced cell migration [143].

The CaMKKβ/CaMK-IV signaling complex also plays an important role in cell migration. In a short commentary by Means [144], he briefly highlights the role of this system in the migration of cerebellar granule cells from the peripheral layer to the internal granular layer of the cerebellum following a brain-derived neurotrophic factor (BDNF) gradient where they connect to Purkinje cells. This was uncovered after knocking-out either CaMKKβ or CaMK-IV, a process that was reverted in vitro by reestablishing the expression of these kinases [145]. This process was initiated by Ca^2+^ influx mediated by the activation of *N*-methyl-D-aspartate (NMDA) receptors, which in turn after Ca^2+^ binding to CaM lead to the activation of the CaMKKβ/CaMK-IV complex. This is followed by autonomously active CaMK-IV entering the nucleus and phosphorylating CREB, which initiates the transcription of the BDNF gene, and hence the production of this neurotrophin required for the directional migration of the granule cells [144]. The migration of neuroblasts to the olfactory bulb is also regulated by CaMK-IV as shown by knocking-down either CaM or this kinase in a transgenic mouse model [146]. The CaMKK/AMPK/Rho/ROCK pathway transduces the Ca^2+^ signal to induce the migration of neurons expressing gonadotrophin-releasing hormone-1 through defined routes traced by axon bundles. This migration mode, denoted axophilic, is believe to be utilized by neurons to reach their destination into the forebrain of developing mice. The involved Ca^2+^-signaling pathway resulted in the formation of actin processes in the leading front edge of the neurons, while actin retraction in the rear end was unaffected by this pathway [147]. Cultured vascular endothelial cells treated with simvastatin, a statin used in the clinic as a cholesterol controlling agent, activate CaMKKβ, which phosphorylates the downstream kinases LKB1 and AMPK (AMP-activated protein kinase), and the latter induces the activation of Rac1 leading to the migratory response of the endothelial cells [148]. The signaling mechanism described in this report was further supported by studies carried out in homogenates of aortic tissue from statin-treated mice [148]. In addition to the role of VEGF-A/B in cell proliferation and the activation of the CaMKKβ/AMPK pathway in aortic endothelial cells, only VEGF-A was shown to stimulate cell migration while VEGF-B did not exert a significant effect [149].

Different Src-family kinases, including c-Src, are involved in the control of cell migration (reviewed in [150,151]). c-Src has been shown to bind and to be strongly activated by both Ca^2+^/CaM and apo-CaM, and strikingly to a greater extent by the latter [152] (reviewed in [153]). This suggests that fluctuations in the cytosolic Ca^2+^ concentration, which change the Ca^2+^-bound/Ca^2+^-free CaM ratio, could modulate c-Src-mediated signaling pathways controlling cell migration. Moreover, c-Src plays a role in cell migration by phosphorylating CaM. Thus, in a model proposed by Chaudhuri et al. [154], activation of this tyrosine kinase by lysophosphatidylcholine results in the formation of phospho-Tyr99-CaM, which binds to the regulatory p85 α-subunit of PI_3_K, releasing its inhibitory action on the catalytic p110 subunit. Wang et al. [155] later showed that binding of non-phosphorylated CaM to the C-terminal SH2 domain of p85 α-subunit led to PI_3_K activation. In addition, since CaM directly binds to p110 with high affinity this might be another way to regulate this lipid kinase [156]. Phosphatidylinositol 3,4,5-trisphosphate, the product of this enzyme, favors the translocation of TRPC6 (transient receptor potential channel 6) to the plasma membrane, its activation, and hence Ca^2+^ entry into the cell, inhibiting the migration of vascular endothelial cells [154]. The authors of this article pointed out that this mechanism appears to play an important role in injured endothelium, as well as in atherosclerosis, because lysophosphatidylcholine is a major component of oxidized low-density lipoproteins present in blood plasma and atheromas. The implication of different phospho-CaM species in a variety of other pathophysiological processes has recently been reviewed [29].

Finally, it is worth mentioning that PDGF-BB-mediated activation of the eukaryotic elongation factor 2 kinase (eEF2K), also named CaMK-III, which is negatively regulated by CaM [157] (reviewed in [158]), enhances the proliferation and migration of VSMCs. This works via the ERK/MAPK, p38MAPK, Akt pathways, and Hsp27 as a downstream substrate of p38MAPK, shown by results using the eEF2K inhibitor A-484954 [159].

### 3.3. The Role of Calcineurin

Calcineurin (CaN) is a Ca^2+^/CaM-dependent phosphatase formed by a catalytic A subunit (CaN-A) and a regulatory B subunit (CaN-B) regulating a large number of cellular functions (reviewed in [160,161]). CaN signals via the transcription factor NFAT. The inhibition of CaN with cyclosporin A and FK506 or the expression of a dominant-negative NFAT mutant inhibits, for example, the migration of a gonadotrophin-releasing hormone secreting neuroendocrine cell line [162]. CaN has been shown to play a role in cell migration related to the turnover of integrins. This was demonstrated in migrating neutrophils, where buffering cytosolic Ca^2+^ or inhibiting CaN induced the accumulation of integrin-α_v_β_3_ at cell adhesions in the trailing end of the cell. These structures got sticky and very strongly attached to the extracellular matrix without easily detachment, because integrins must be recycled back from the rear end to the leading front of the cell to proceed the migration [163]. 

### 3.4. CaM-Regulated Small G Proteins

Small GTP-binding proteins, also denoted small G proteins, are 20–40 kDa GTPases that participate in many signaling pathways controlling a plethora of cellular functions (reviewed in [164,165,166]). Interestingly, a protein denoted Trio has two guanine nucleotide-exchange factor (GEF) domains, one specific for Rac and the other for Rho, and a protein kinase domain similar to the one in CaMK. This protein was shown to associate with the transmembrane tyrosine phosphatase denoted leukocyte common antigen-related (LAR), also known as protein tyrosine phosphatase receptor F (PTPRF) [167], which is located close to and regulates the function of focal adhesions via cyclin-dependent kinase 1 [168]. This suggested to the authors of this work that Trio may coordinate functions required for cell migration such as cytoskeletal and cell-matrix remodeling, acting as a multidomain signaling platform [167].

Small G proteins belonging to the Rho and Rap families play essential roles in cell migration. Within the Rho family, RhoA regulates the formation of actin-myosin stress fibers and focal adhesions. Rho-kinase (ROCK) intervenes in this process as a downstream effector by phosphorylating myosin light-chain inducing actomyosin contraction (see Figure 2). On the other hand, Rac1 induces actin polymerization and is implicated in the formation of lamellipodia, peripheral ruffles, and focal complexes. The latter are very dynamic structures in the leading edge of migrating cells that will be transformed into more stable focal adhesions during migration. It is interesting to consider that GTPase-activating protein (GAP) members containing a specific G-actin recognition sequence bind to and are inhibited by the pool of free soluble G-actin. This results in higher levels of active GTP-loaded Rac and Cdc42 which favor the polymerization of G-actin into actin filaments [169]. Rac1 also activates p21-activated kinase (PAK), a downstream signaling kinase that phosphorylates paxillin, which is a signal transduction adaptor protein located at focal adhesions. Finally, Cdc42 as Rac1 induces the formation of peripheral ruffles and focal complexes as well as the formation of filopodia and uses myotonic dystrophy kinase-related Cdc42-binding kinase (MRCK) as a downstream kinase for signaling. The Rap GTPase family has been implicated as well in directional migration of vascular endothelial cells and lymphocytes, and in fibroblasts Rap activation induces the activation of Rac. For detailed reviews about the mechanistic role of different small G proteins, the downstream kinases ROCK, PAK, and MRCK, and other key systems implicated in cell migration in normal and cancerous cells see [170,171,172,173,174,175,176,177].

Rac1 and Cdc42 have been shown to directly interact with CaM in a Ca^2+^-dependent manner, and their highly similar CaM-binding sites were identified [178,179]. However, while binding of Ca^2+^/CaM to Rac1 induced its activation, Cdc42-Ca^2+^/CaM binding had an opposite effect [178]. This study was conducted in human platelets and it remains to be investigated whether CaM may also modulate the dynamics of the actin cytoskeleton during cell migration in other cell types in a similar manner. In fact, activation of Rac1 with thrombin or EGF in HeLa and megakaryoblastic leukemia cells was demonstrated to be dependent on CaM binding to the GTPase. This was partially aborted by mutating the CaM-binding site, and CaM overexpression or downregulation with shRNA altered Rac1 activation, a process that was also observed in the absence of EGF stimulation [84]. In this study, W-7 also inhibited Rac1 activation, and no interaction of RhoA or H-Ras with CaM was detected, underscoring the specificity of the Rac1/CaM interaction. In contrast, K-Ras bound and was regulated by CaM [180,181]. Stimulation of fibroblasts with PDGF enhanced the interaction of Ca^2+^/CaM with K-Ras and lead to Akt activation and cell migration, a process that was inhibited by the CaM inhibitor *N*-(4-aminobutyl)-5-chloro-2-naphthalenesulfonamide (W-13) [182]. Furthermore, in a megakaryocyte cell line, thrombin and collagen activated Rac1 and Cdc42, but W-7 only prevented Rac1 activation while leaving Cdc42 activation unaffected [85]. In this study, it was also demonstrated that transient overexpression of CaM enhanced Rac1 but not Cdc42 activation. Overall, this suggests that only Rac1 is positively regulated by CaM in agreement with previous studies in platelets [178].

**Figure 3:**
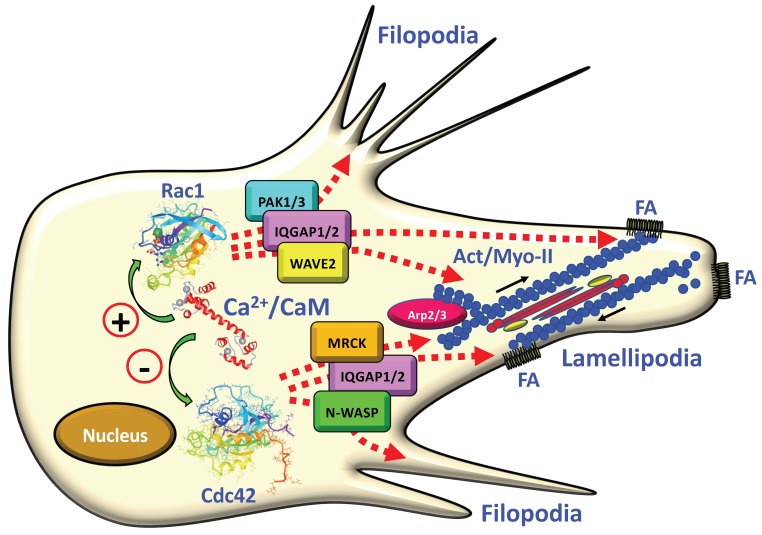
**CaM-regulated small GTPases and cell migration.** The small G proteins Rac1 and Cdc42 are Ca^2+^/CaM-binding proteins that intervene in cell migration and control one or more of the following processes: the polymerization of actin-stress fibers, the assembly of focal adhesion plaques, and the protrusion of filopodia, lamellipodia, and peripheral ruffles. Rac1 is activated by Ca^2+^/CaM while Cdc42 is inhibited by this regulator. The downstream kinases MRCK and PAK1/2, the scaffold proteins IQGAP1/2 and the activators of the Arp1/2 complex WAVE2 and N-WASP are depicted. Rac1 and Cdc42 interact with and activate these proteins. The structures of human Ca^2+^/CaM (ID: 1CLL) [40], human Rac1 (3TH5) [189], and human Cdc42 (1AJE) [190], were obtained from the Protein Data Bank. The structures of the G proteins are shown in a rainbow color code (N-terminus, purple and C-terminus, red). Rac1 is depicted bound to the GTP analogue phosphoaminophosphonic acid-guanylate ester. Filopodia, lamellipodia, focal adhesions (FA) and actomyosin-stress fibers (Act/Myo-II) are also depicted. Dashed red arrows point to structures regulated by Rac1/Cdc42. Arp2/3, actin-related proteins 2/3; IQGAP1/2, IQ motif containing GTPase activating proteins 1/2; MRCK, myotonic dystrophy kinase-related Cdc42-binding kinase; PAK1/3, p21-activated kinases; N-WASP, neural Wiskott–Aldrich syndrome protein; WAVE2, WASP family verprolin-homologous protein. See text and references [170,171,172,173,174,175,176,177,178,179] for more details.

Figure 3 depicts the interaction of Ca^2+^/CaM with Rac1 and Cdc42 inducing the activation of the former and inhibition of the latter. In a very simplified manner the action of these small GTPases on the protrusion of filopodia and lamellipodia by regulating polymerization and contraction of actomyosin stress fibers, and the assembly of focal adhesions is highlighted. The signaling mechanisms playing key roles in regulating Rac1/Cdc42 downstream activities involve the effector kinases PAK1/3 and MRCK (reviewed in [173,183]), the scaffold proteins IQGAP1/2 [184,185], and the Wiskott–Aldrich syndrome type proteins WAVE2 (WASP family verprolin-homologous protein 2) and N-WASP (neural Wiskott–Aldrich syndrome protein), the latter two participating in activation of the actin-binding complex Arp2/3 (actin-related proteins 2/3) [186] (reviewed in [187]). It is of interest to consider that small GTPases not only send signals when anchored at the plasma membrane but also when located at endocytic compartment membranes (reviewed in [188]).

As RhoA does not interact with CaM, construction of a chimera protein with an inserted natural or synthetic CaM-binding peptide, an IQ motif and CaM in this GTPase allowed the control of RhoA activity in response to the generation of Ca^2+^ signals depending on the inserted peptide [191]. Hence, this synthetic biosystem could be used for reversible Ca^2+^-mediated regulation of cell morphology leading to cell migration. This is due to the movement of CaM from the IQ motif, where it is bound as apo-CaM when the Ca^2+^ concentration is very low, to the MLCK Ca^2+^/CaM-binding site insert, when the concentration of Ca^2+^ rises. Similarly, Rac1-CaM and Cdc42-CaM chimeras containing CaM-binding sensor peptides to induce structural changes upon Ca^2+^ binding to the CaM moiety, and yellow fluorescent protein for visualization, have been engineered. These fusion proteins directly respond to Ca^2+^ influx activating the GTPase module, which induces lamellipodia and filopodia growth. Combining in the same cell one of these constructs with another blue-light sensitive Ca^2+^-mobilizing construct that opens Orai1 channels, previously developed by the same authors, induced the described cellular responses upon illumination with a blue light source [192].

In addition to the regulation of some small G proteins by CaM, there are GEFs which interact with and are regulated by CaM. One example is IQSec/BRAG, a GEF of the Arf family that is implicated in metastasis in different kinds of cancers. It has an IQ motif located at its N-terminus and is atypical for most CaM-binding proteins, as it binds Ca^2+^-free CaM (apo-CaM) and releases CaM upon Ca^2+^ binding (reviewed in [193]).

## 4. Calmodulin-Regulated Proteins in Cell Invasion and Metastasis

The multistage metastatic process involves a complex set of events by which a subset of highly malignant cells from the primary tumor are disseminated and established in distant organs, which results in the high mortality rate of oncology patients (reviewed in [194]). Tumor migrating cells form either trails of clustered blazer cells, or are maintained clustered in an opportunistic manner by mutual stimulation by tumor and normal cells-derived extrinsic factors (reviewed in [195]). The acquisition of metastatic properties by tumor cells is controlled by a set of different programs encompassing epithelial-masenchymal transition (EMT), by which epithelial cells acquire mesenchymal phenotype. CaMK-D was shown to inhibit EMT via the transcription factor Snail, as well as cytoskeleton remodeling, and to promote the migration and invasiveness of a variety of tumor cells, while different CaMK-D isoforms may exert opposite or identical effects on the progression of different kind of tumors, such as prostate, breast, skin, pancreatic, and gastric carcinomas (reviewed in [196]). Small GTPases also play a fundamental role in tumor cell invasiveness and metastasis development. In this context, the synergistic effect of Rho and Rac family members control EMT induced by TGF-β and subsequent tumor cell motility, suggesting that these GTPases could be useful therapeutic targets against the spread of cancer (reviewed in [197]). Moreover, it has been established that shifting melanoma cell migration from an amoeboid mode, primarily mediated by Rho/ROCK signaling and characterized by high actomyosin contractility, to a mesenchymal-type mode, mediated by Rac1/WAVE2 signaling and characterized by low actomyosin contractility and high proteolytic activity, is an interconvertible mutually exclusive process regulated by a Rac1 activation/inactivation cycle [198].

Multiple factors are involved in the preparation of niches in the metastatic foci, where effectors supplied by exosomes also play a significant role in the metastatic process (reviewed in [199,200,201]).

An interesting aspect of tumorigenesis is the occurrence of tumor-normal cells competition, by which the more competent cells eliminate the less competent ones. This occurs in both directions: normal cells acting as tumor suppressors can eliminate tumoral ones as a defense mechanism, and tumor cells acting as super-competitors usually outcompete normal cells, eliminating them by engulfment or by inducing apoptosis, among other mechanisms, favoring therefore tumor progression and metastasis development (reviewed in [202]). In host-tissues, where mesenchymally-transformed tumor cells migrate to form metastasis foci, a mesenchymal-to-epithelial transition (MET) usually occurs (reviewed in [203]).

As counterparts of podosomes in normal cells, in tumor cell invadopodia/invadosomes are essential structures that explain the high invasive capacity of metastatic cancerous cells. Invadosomes allow the penetration of tumor cell protrusions into the extracellular matrix and the basal lamina of vessels, inducing their proteolytic degradation by activated metalloproteases facilitating in this manner invasiveness. Detailed reviews about the structure and function of invadosomes have recently been published [204,205,206].

A recent exciting discovery that helps to explain the progression of primary and metastatic brain tumors is the finding that tumor cells establish synaptic connections with glutamatergic neurons, and that neuronal firing stimulates the tumor cells by allowing Ca^2+^ entry caused by the expression of NMDA and AMPA (α-amino-3-hydroxy-5-methyl-4-isoxazolepropionic acid) receptors in the tumor cells. The inter-cellular transmission of the Ca^2+^ signal among the network of apposed tumor cells is propagated via gap junctions. This has been demonstrated not only in gliomas but also in brain metastasis from breast carcinomas [207,208,209] (commented in [210]). Inter-cellular Ca^2+^ signaling enhances tumor cell proliferation, migration, and invasiveness as well as the colonization of the brain with metastatic foci, processes involving multiple Ca^2+^/CaM-regulated proteins, and contributing to enhance patient lethality as discussed below.

Another process of great importance in the establishment of the metastatic foci is tumor-associated angiogenesis, a process in which different cytokines and growth factors play an essential role (reviewed in [211,212]). It has been suggested that the anti-angiogenic activity of 2-methoxyestradiol was due to its antagonistic action on CaM by blocking cell cycle progression in metaphase [213]. Calponin-h1, a CaM-binding protein that interacts with actin and tropomyosin, is involved in the formation of stress fibers and smooth muscle contraction. Interestingly, this protein is a tumor suppressor with angiogenic properties, as demonstrated in rat 3Y1 cells transformed with *v-src* by reducing VEGF expression, and hence cell proliferation and cell motility [214]. Finally, in connection with the function of CaM in angiogenesis it is worth mentioning that the anti-angiogenic action of TNF-α is due to FMRP (fragile X chromosome mental retardation protein) dephosphorylation, facilitating in this manner the expression of miR-181a, a microRNA that blocks CaM translation, therefore preventing CaMK-II activation [215].

### 4.1. CaM-Dependent Protein Kinases

The implication of CaM-dependent protein kinases in invasiveness and the metastatic capacity of tumor cells is well known. Here, we will discuss several examples where CaMKK, CaMK-I, CaMK-II, DAPK (death-associated protein kinase), CASK (Ca^2+^/CaM-activated serine kinase), and eEF2K (CaMK-III) are implicated in these processes. For a recent review on the role of different CaM-dependent kinase family members in tumor cell invasiveness and their consideration as potential therapeutic targets see [216].

For the activation of CaMKs by CaM a surge in intracellular Ca^2+^ concentration, mediated by Ca^2+^ channels, is required for the initial formation of the Ca^2+^/CaM complex. An example documenting the importance of Ca^2+^ mobilization in this context is Ca^2+^ influx through the Orai channel/STIM system, activating CaMK-II and leading to enhanced tumor cells invasiveness and metastasis. This was concluded based on the observation that downregulating Orai or STIM by shRNAs or CaMK-II inhibition suppressed the CaMK-II/MAPK signaling pathway, resulting in the inhibition of human melanoma cells migration and metastasis in the lungs [217]. For a detailed review on the role of the Orai/STIM system in tumor cells invasiveness and the development of metastasis, see [218], and for a review on the role of CaMK-II in cellular scattering, and actin cytoskeleton dynamics leading to the rupture of cell–cell adhesion as a key feature of EMT, see [132].

Medulloblastoma is a highly metastatic neural tumor, which tends to infiltrate meningeal tissues resulting in neoplastic meningiosis in children. The migratory capacity of medulloblastoma cells is regulated by the CaMKK/CaMK-I pathway and the small G protein Rac1, as well as its GDP/GTP-exchange factor GEF/Rac1 [219]. CaMKKβ is an upstream component of the AMPK signaling pathway participating in cell migration initiated by Ca^2+^-mobilization through the activation of the lysophosphatidic acid receptor in ovarian cancer cells [220]. This is an example of the importance of CaM in activating kinases by the transient increase in cytosolic Ca^2+^ concentration induced by a great variety of receptors, including GPCRs. Prostate carcinoma cells have a preference to form bone metastasis driven by the androgen receptor, which induces the expression of CaMKK2 as its key downstream signaling protein playing a fundamental role in tumor cell migration and invasiveness via the CaMKK2/AMPK pathway [221]. Based on these findings it has been suggested that this signaling route could be a potential therapeutic target (reviewed in [222]). Furthermore, direct interaction of the tumor cells with osteoblasts occurs, and CaMKK2 in osteoblasts, osteoclasts, and macrophages signals to remodel the bone tissue after exerting multiple actions affecting the microenvironment that facilitate the establishment of metastatic foci (reviewed in [223]). Gene amplification and overexpression of CaMK-1D frequently occurs in triple(ErbB2/estrogen/progesterone receptors)-negative human breast carcinoma basal-like subtype, and is associated with increased invasiveness [224]. To corroborate these findings on a functional level the authors of this study demonstrated that forced expression of CaMK-1D in non-tumorigenic mammary epithelial cells induced increased proliferation, epithelial–mesenchymal transition, loss of cell–cell attachment, and increased migratory capacity, processes leading to invasiveness [224].

Hepatocellular carcinomas show increased expression of the transcriptional regulator Yap, which contributes to cell migration and invasiveness. The molecular mechanism by which Yap promotes this outcome has been established using liver carcinoma cells lacking Yap expression by stable shRNA-transfections [225]. It was shown in this cellular system that the absence of Yap promotes the expression of Bnp3, a protein that induces mitophagy and mitochondrial failure, depleting the ATP pool of the cell with subsequent failure of the sarco-(endo)-reticulum Ca^2+^-ATPase. This resulted in cytosolic Ca^2+^ overload, inducing CaMK-II activation leading to cofilin phosphorylation that blocked the polymerization of actin filaments. Polymerized actin is required for the formation of protruding lamellipodia needed for tumor cell migration [225]. Mitochondria CaMK-II (mtCaMK-II) also contributes to cell migration. Phosphorylation of the mitochondrial Ca^2+^ uniport (MCU) at Ser92 by mtCaMK-II enhances Ca^2+^ entry into the mitochondrial matrix, which is required for CaMK-II activation, closing this positive feedback mechanism. Thus, the inhibition of mtCaMK-II or MCU inhibits PDGF-induced VSMC migration and the translocation of mitochondria to the leading migratory edge of the cell, decreasing therefore FAK phosphorylation, focal adhesion turnover, and the necessary remodeling of the cytoskeleton, processes required for cell migration [226] and likely occurring as well in tumor cells.

CaMK-II plays as well an important role in metastasis. Thus, knocking-out the CaMK-II gene in a highly metastatic prostatic cell line inhibited cell migration and metastasis development [227]. This has been confirmed in vitro using cultured human colon cancer cells by inhibiting CaMK-II with a chemical inhibitor leading to inhibition of cell migration and invasiveness [228]. The mucin-like protein AGR2 (anterior gradient 2) that is present in mucus-secreting tissues as, for example, in the colon, is secreted and able to activate a non-canonical Wnt pathway implicating Wnt11 triggering activation of the CaMK-II and JNK pathways, which jointly activate the migration and invasiveness of colon carcinoma cells required for metastasis development [229]. Moreover, enhanced expression of CaMK-II in human breast cancer cells, as compared to normal breast tissue, and its activation by CaM leading to autophosphorylation at T286 positively correlated with increased invasiveness, anchorage-independent growth and lower metastasis-free survival rate. Furthermore, transfection with the phospho-mimetic T286D CaMK-II mutant plasmid, rendering this enzyme in a constitutive active form, resulted in FAK phosphorylation, which further enhanced the migratory and invasive capacity of the tumor cells, suggesting that CaMK-II plays a key role in metastasis [230]. The authors of this report proposed that targeting CaMK-II could be a good choice to inhibit breast cancer metastasis. Metastatic gastric carcinoma cells also exhibit enhanced expression of CaMK-II which correlates with their increased migration and invasive capacities [231]. Screening a library of protein kinase inhibitors revealed that CaMK-II inhibitors, among inhibitors for other kinases, attenuated breast cancer stem cell migration and EMT, which are important processes in metastasis development [232]. Furthermore, downregulation of CaMK-IIα with shRNA or its overexpression, respectively induced the inhibition or the acceleration of the rate of proliferation, migration and invasiveness of transfected human osteosarcoma cell lines. These results obtained in vitro were corroborated in vivo by implanting these tumor cells in the tibia of immunodeficient athymic mice [233].

The expression of the physiological inhibitor-α of CaMK-II in primary medullary thyroid carcinoma cells inversely correlates with the capacity to form lymph node metastasis [234]. In human follicular thyroid cancer cells Ca^2+^ entry via TRPC1 (transient receptor potential channel 1) appears to be important for migration and invasiveness. The evidence for this is based in the observation that knocking down this channel downregulated the expression of the receptors for sphingosine-1-phosphate and VEGF and the migration induced by these factors. The mechanism implicates CaM and CaMK-II since the expression of both receptors was blocked with W-13 and KN-93, inhibitors of CaM and CaMK-II, respectively [235]. The authors of this study suggested that the expression of this inhibitor could be a good marker of tumor aggressiveness and metastatic dissemination.

Glioblastomas are highly malignant brain tumors that very rarely form distant metastasis but have an extraordinary local infiltrating and invasive capacity. The invasiveness of glioblastoma cells is to a great extent due to the existence of glioblastoma stem cells in the primary tumor with high migratory capacity [236]. High levels of CaM in these tumors play a critical role in activating invadopodia-associated proteins [237]. In agreement with this, W-7 inhibited invadopodia formation and hence the migration of the breast tumor cell line Met-1 [238]. CaMK-IIγ has been implicated in the signaling pathways responsible for the stemness of glioblastoma cells involving the activation of a series of transcription factors including Nanog, Sox2 and Oct4. The CaM inhibitor hydrazinobenzoylcurcumin has anti-tumor effects by inhibiting, among other processes, the migratory and the invasive capacity of glioblastoma stem cells as a result of targeting the CaMK-II/c-Ret pathway [239]. c-Ret is a tyrosine kinase receptor activated by glial-derived neurotrophic factor (reviewed in [240]). Furthermore, curcumin also suppressed the activation of the transcription factor Sp-1 downregulating in a concentration dependent manner Sp-1-dependent transcription of a series of target proteins, including CaM, which are implicated in invasiveness and metastasis [241].

The high infiltrating capacity of glioblastoma multiforme in surrounding brain tissues is explained in part by the activation of the voltage-regulated chloride channel CIC-3 and the Ca^2+^-dependent potassium channel K_Ca_3.1, which regulate cell volume by the exit of intracellular Cl^-^ and K^+^. The subsequent loss of water produces cell shrinkage facilitating their migration through narrow interstitial spaces [242,243]. The phosphorylation of CIC-3 by CaMK-II has been shown to activate this channel. Therefore, inhibition and/or knocking down either CaMK-II or CIC-3 decreased the migration of these cells [242,243]. Similar results, showing that the acid-sensitive non-selective transient ion channel ASIC1a interacts with and is phosphorylated by CaMK-II regulating its activity and controlling glioblastoma cell migration, have been obtained [244]. The combined regulation of CIC-3 and ASIC1a by CaMK-II could be a key feature for net cell volume change during cell migration. Furthermore, the role of CaMK-II regulating CIC-3 and the implication of this system in cell migration was also determined in VSMCs [245].

Dephosphorylation of Ca^2+^/CaM-dependent kinases occurs via Ca^2+^/CaM-dependent protein kinase phosphatases (CAMKP and CAMKP-N), which play a relevant role in tumor cell biology, including metastasis development among other processes (reviewed in [246]). For example, higher expression of an isoform of CAMKP-N (also denoted POPX2) in breast cancer increases the invasiveness and incidence of tumor metastasis during the early stages of the illness but plays an opposite role at latter stages, as increased invasiveness was demonstrated by knocking down this phosphatase in a human breast carcinoma cell line [247]. In addition, it was shown that miRNA-149 directly targeted CAMKP (also denoted PPM1F) in hepatocellular carcinoma (HCC) and downregulation of this microRNA positively correlated with metastasis development. On the contrary, the forced expression of miRNA-149 inhibited cell migration and tumor cell invasiveness [248]. The authors of this report suggested to use miRNA-149 as a potential biomarker of HCC and that CAMKP could be a novel target to combat HCC metastasis. Overall, these results underscore the relevance of CaMK-II autoactivation and inactivation by specific phosphatases in the development of metastasis.

Death-associated protein kinase (DAPK) is a Ca^2+^/CaM-dependent protein kinase with tumor suppressor properties implicated in apoptosis, autophagy, immune response, and inflammatory processes with high relevance for cancer biology (reviewed in [249,250]). There is an inverse correlation between DAPK expression in murine lung carcinoma cells and their metastatic capacity, underscoring the tumor-suppressive properties of this kinase [251]. Furthermore, these authors demonstrated that restoring DAPK expression in the highly-metastatic Lewis carcinoma cell line suppressed its capacity to form lung metastasis. They found that this process was due to the DAPK-mediated induction of tumor cell apoptosis, and by this linking suppression of apoptosis to metastasis. This is correlated with the inhibitory effect that DAPK exerts on Cdc42 and integrin-β_1_ activation, preventing therefore the required polarization during the directional migration of tumor cells [252].

In metastatic pituitary tumors it was shown that the methylation or deletion of CpG islands in the promoter of the DAPK gene repressed the expression of this kinase, and that this was positively correlated with a higher rate of metastasis development [253]. In cholangiocarcinoma, however, although aberrant hypermethylation of the DAPK promoter was detected in 11 of 36 cases, no significant difference in invasion and lymph node metastasis was found as compared to cases where no hypermethylation was detected [254]. Loss or failure in the recruitment of the multi-subunit Mediator complex for RNA polymerase II-dependent gene transcription Med1 and/or the transcription factor C/EPB-β (CCAAT/enhancer binding protein-β) to the CRE/ATF (cyclic-AMP response element/activating transcription factor) site of the *DAPK* gene promoter appears to be responsible for the loss of DAPK expression in human lung and breast adenocarcinoma cell lines. This could be corroborated by showing that restoration of Med1 yielded DAPK1 expression, which decreased the development of metastasis in vivo [255]. DAPK is synergistically regulated by phosphorylation and dephosphorylation of residues Tyr491/Tyr492 by c-Src and the tyrosine phosphatase LAR, respectively. EGF-induced c-Src activation promoted an early phase of DAPK phosphorylation and a moderate inactivation of its activity, while the subsequent dephosphorylation of DAPK by LAR enhanced its activity promoting apoptosis and the anti-adhesion and anti-migratory properties of this kinase. However, since at a later phase c-Src induces the downregulation of LAR expression, the combined action of both apparently antagonist systems further reduced net DAPK activity, enhancing therefore EGF-dependent tumor cell migration and invasiveness [256].

Another CaM-dependent kinase is the highly conserved Ca^2+^/CaM-activated serine kinase (CASK). This multi-domain kinase, belonging to an extensive group of membrane-associated guanylate kinase proteins with scaffolding properties, was first found to play an important role in the development of the neural system but plays additional roles in cancer progression (reviewed in [257,258]). Overexpression of CASK together with the heparan sulfate proteoglycan syndecan-2 was associated with the higher metastatic capacity of colorectal cancers affecting lymph nodes, increased vascular invasiveness, and liver metastasis [259]. Interestingly, it has been shown that miRNA-203 plays a role in preventing *Helicobacter pylori*-induced gastric carcinoma, and that the suppressive function of this microRNA blocking invasiveness is due to the downregulation of CASK, while overexpression of this kinase rescued the suppressive effect of miRNA-203 [260].

The Ca^2+^/CaM-dependent kinase eEF2K/CaMK-III is overexpressed in triple-negative human breast carcinomas. Expression of this kinase is downregulated by miRNA-603, which resulted in inhibition of cell migration and the invasiveness of these tumor cells, inhibiting as well cell proliferation, angiogenesis and inducing apoptosis [261]. Consistent with this, downregulation of eEF2K with siRNA or rottlerin in a highly metastatic ductal pancreatic carcinoma cell line inhibits invasiveness and EMT by decreasing the expression of transglutaminase as well as integrin-β_1_, urokinase receptor (uPAR), and matrix metalloprotease-2 (MMP-2), and decreasing the activity of c-Src, while overexpression of eEF2K enhanced the migratory and invasive capacity of the tumor cells [262]. It was demonstrated that rottlerin inhibits the migration and invasiveness of two human glioma cell lines, along with its action arresting the cell cycle and inducing apoptosis, and in addition to targeting eEF2K also targets the cell cycle regulator Cdc20 [263]. This suggests that not all actions of this compound are due to eEF2K inhibition.

Serine/threonine-protein kinase 33 (STK33) belongs to the Ca^2+^/CaM-dependent kinase family [264], although to the best of our knowledge, no information on the direct activation of STK33 by CaM is available. Increased methylation of the STK33 gene promoter and decreased protein expression was observed in human colorectal carcinoma samples as compared to normal surrounding tissue. This was positively correlated with increased infiltration, lymph node invasion, and distant metastasis [265], suggesting that STK33 activity could have metastasis-suppressive properties similar to the case of DAPK1 [255].

### 4.2. Calcineurin

Calcineurin controls signaling pathways relevant for the migration, invasiveness, and metastatic capacity of tumor cells. The CaN/NFAT signaling pathway plays a prominent role in the development of metastasis as demonstrated in a cohort of clinical cases of human breast cancer, particularly in triple-negative tumors, as confirmed by downregulating CaN, NFAT1 and NFAT2 with shRNAs in a murine tumor cell line [266]. A positive correlation between NFAT1 expression and metastasis in non-small cell lung carcinoma (NSCLC) was demonstrated in a set of clinical cases [267]. In addition, the calcineurin inhibitors FK506 and cyclosporin A (CsA) were shown to prevent the phosphorylation of FAK and paxillin, and the attachment of non-adherent colon carcinoma cells induced by protein kinase inhibitors, a process that was also blocked by the CaM inhibitors W-7 and calmidazolium [70].

The action of CsA on invasiveness and tumor metastasis is complex and distinct in different tumor cells. In adenocarcinoma cells, it was shown that CsA enhanced cell motility, anchorage-independent growth, and metastasis development in a TGFβ-dependent manner, and it was prevented using anti-TGFβ monoclonal antibodies [268]. Furthermore, in addition to the effect observed in these tumor cells, CsA was able to induce invasiveness of non-transformed cells [268]. These findings call for words of caution and close surveillance of transplanted patients treated with this immune suppressive agent. In contrast, glioblastoma cell invasiveness has been shown to be enhanced by microglia and it was inhibited by CsA [269]. Human breast carcinoma cells with increased inducible expression of COX-2 have enhanced invasiveness, which was inhibited by COX-2 inhibitors and by CsA [270]. Some unknown factors must be responsible for the opposed effects of CsA observed in different tumor cell types, but this is apparently not dependent on the tissular origin of the tumor. This is documented in two highly metastatic human melanoma cell lines, HT168 and WM35, in which CsA respectively enhanced and inhibited fibronectin-guided cell migration, a process that is accompanied by a CsA triggered switch in the expression of integrin-β_3_ to the β_1_ isoform in HT168 cells but not in WM35 cells [271].

Overexpression of CaN has been observed in highly metastatic small-cell lung carcinomas (SCLC) where bone metastasis occurs, in contrast to those tumors where bone metastasis was absent. Moreover, it was observed that the occurrence of metastasis in the former tumors was concomitant with the location of CaN in the nucleus, while in the non-metastatic tumors CaN was mainly located in the cytosol [272]. The decreased expression of the catalytic CaN-Aα subunit in SCLC by a lentiviral vector-induced RNA interference method inhibited, among other processes, the migratory and invasive capacity of the tumor cells as well as their adhesion to the bone matrix, hampering metastasis development [273]. Interestingly, although overexpression of the regulatory CaN-B subunit did not enhance the net activity of CaN in transfected cells, their migratory capacity increased, as well as cell proliferation and anchorage-independent cell growth. This was correlated with an independent oncogenic property of CaN-B, which is unrelated to activation of the phosphatase [274].

### 4.3. CaM and Matrix Metalloproteases

Matrix metalloproteases (MMPs) play a critical role in the disruption of the basal membrane and degradation of the surrounding connective tissue extracellular matrix during the dissemination of tumor cells and the establishment of distant metastatic foci, in addition to other functions relevant for tumor progression (reviewed in [275,276,277]). Due to their great importance during metastasis development, targeting these proteases has been considered an important objective of anti-tumoral therapy, resulting in the development of a great variety of MMP inhibitors (reviewed in [278]). In this context, it has been shown by ingenuity pathway analysis that the BDNF/TrkA (tropomyosin receptor kinase A) pathway regulates the network of MMPs and CaM in triple-negative human breast carcinoma cells modulating the interaction of tumor cells with endothelial cells [279]. HRPAP20 (hormone-regulated proliferation-associated protein 20) is a CaM-binding proliferation-associated protein highly expressed in invasive breast cancer cells and is implicated in matrix metalloprotease-9 (MMP-9) secretion [280]. HRPAP20-transfected tumor cells further increased their original invasive capacity, while knocking-down its expression resulted in reduced invasiveness. CaM binding to HRPAP20 was shown to be essential for this process as mutation of its CaM-binding site inhibited MMP-9 secretion [280]. Expression of MMP-9 in human breast cancer cells and cell migration have been shown to be induced by the Ca^2+^/CaM-binding protein TBC1D3, and these effects were enhanced upon Ca^2+^/CaM binding to this protein, as it prevented its ubiquitination and degradation [281]. In addition, CaMK-II-mediated upregulation of the transcription of NFκB, which is critical for MMP-9 expression, contributed to the invasiveness of gastric carcinoma cells [231]. Nagano and collaborators [282] demonstrated that Ca^2+^ influx induced the interaction of Ca^2+^/CaM with the metalloprotease ADAM10, inhibiting its activity, while the CaM antagonist trifluoperazine facilitated the conversion of the inactive pro-form of the protease to its active form, resulting in increased proteolysis of the adhesion molecule CD44. In this context, it was shown that high-grade pituitary adenomas overexpressed ADAM10, as compared to the low-grade tumors, and this positively correlated with their higher migratory capacity and invasiveness. This is due in part to the ADAM10-mediated cleavage of the ectodomain of CD44 caused by a reduced binding of the protease with CaM [283].

### 4.4. CaM-Regulated Scaffold/Adaptor Proteins

The adaptor protein Grb7 binds CaM in a Ca^2+^-dependent manner [284], as this is also the case for the Grb7 family members 10 and 14 [285], and plays an important role in cell migration and tumor cell invasiveness (reviewed in [21,286,287]). Deletion of the CaM-binding site of human Grb7, located in the proximal region of its pleckstrin homology domain, had an inhibitory effect on cell attachment to the substrate, impaired the migratory capacity of the cells and the development of brain tumors in vivo shown by implantation of rat glioblastoma cells transfected with a Grb7-mutant lacking the CaM-binding site [288,289]. This later effect cannot be ascribed only to the impaired invasiveness, as deletion of the CaM-binding site also inhibited cell proliferation and tumor-associated angiogenesis [289].

The scaffold protein IQGAP1 binds CaM in Ca^2+^-dependent and Ca^2+^-independent manners via a set of distinct IQ motifs. This protein interacts with actin by acting as a barber end capper [290,291], and intervenes in cytokinesis involving the Rho family GTPases Cdc42 and Rac1 in a CaM-dependent manner [292,293,294]. Similarly, IQGAP2 binds CaM via its four IQ motifs, interacts with actin, Cdc42 and Rac1, but not with RhoA, inhibiting their activation by preventing interaction with RhoGAPs [295]. Overall, these findings implicate IQGAP proteins in the formation of cytoskeletal actin filaments in the protruding leading edge of migrating cells (see Figure 3). Different IQGAP isoforms interact differentially with a variety of target proteins implicated in tumor cell invasiveness and metastatic capacity (reviewed in [296]). In fact, IQGAP1, but not IQGAP2, also participated in the retraction of lamellipodia, decreasing the number of adhesion points most likely upon CaM interaction [297]. Non-invasive MCF-7 human breast adenocarcinoma cells expressing mutated IQGAP1 affecting the Ca^2+^/CaM-binding but not apo-CaM binding sites increased the rate of cell migration [298]. This demonstrated a differential role of Ca^2+^/CaM and apo-CaM on IQGAP1-mediated cell migration. In addition, upregulation of IQGAP1 enhanced the Wnt/β-catenin signaling pathway in pancreatic ductal adenocarcinoma cells inducing cell migration, invasiveness, and EMT, while its downregulation hindered these processes [299]. As previously mentioned, EMT is a complex cellular process, by which epithelial cells detach from each other and from the basal membrane and acquire mesenchymal characteristics by activation of specific transcriptional programs, and this has important physiological relevance during embryogenesis, tissue repair, and wound healing. Its dysfunction also plays an important role during invasiveness and metastasis development, processes where cells showing an intermediate epithelial–mesenchymal state with tumor stem cell characteristics have been identified (reviewed in [300]).

AKAP12, which has four CaM-binding sites, plays a scaffolding role by binding different signaling proteins including CaM, coordinating several cellular signaling pathways involved for example in mitogenesis and cytoskeletal remodeling, and has a metastasis suppressive role (reviewed in [301]). Therefore, AKAP12 downregulation caused by methylation of its promoter or gene loss is associated with enhanced metastatic capacity, and conversely its forced re-expression in tumor cells suppresses cell motility and invasiveness (reviewed in [301]). This effect is most likely not only due to the CaM-binding capacity of AKAP12 but to a combination of other signaling molecules known to interact with this multi-domain scaffold protein.

### 4.5. Other CaM-Binding Proteins

The Na^+^/H^+^-exchanger 1 (NHE1) binds Ca^2+^/CaM at its C-terminal region preventing auto-inhibition, and upregulation of this transport system plays an important role in EMT and metastasis development in triple-negative human breast carcinoma, a process that is inhibited by a specific NHE1 inhibitor [302,303]. CaM plays an important role in this process, as cells expressing a NHE1 with a mutation of the CaM-binding site that yields a constitutive active protein, showed enhanced migration, invasion, and spheroid growth, suggesting an increase of the metastatic potential of these carcinomas [302]. In human hepatocarcinoma cells it was demonstrated that IL-6 induces cell migration and invasiveness, mediated by facilitating the interaction of NHE1 and CaM, forming a molecular complex with enhanced transport activity that regulates intracellular pH and Ca^2+^ concentration [304]. In contrast to what happens in other cells, stimulation of NHE1 by angiotensin-II, due to cytosolic Ca^2+^ increase and subsequent CaM activation, induced the repression of melanoma cell migration, a process that was mirrored by losartan, an inhibitor of the angiotensin-II receptor type 1 (AT_1_) while promoting the adhesion and invasiveness of these tumor cells [305].

## 5. Targeting Calmodulin-Dependent Systems to Inhibit Tumor Cell Invasion and Metastasis

Inhibition of the invasive and metastatic capacity of cells derived from solid tumors is of great clinical interest, as these processes are the main cause of oncological patient mortality. Recently, Gandalovicová et al. have introduced the term migrastatics to define a series of drugs that block different types of tumor cell invasive modes targeting the most downstream signaling systems of the motility machinery (reviewed in [306]). These drugs mainly target systems implicated in actin polymerization and actomyosin contractility, including, for example among many others, WASP family members and cofilin amid the former group, and MLCK, ROCK, and MRCK among the latter one (see Figure 2 and Figure 3). Cytostatic anti-proliferative agents and drugs targeting upstream systems also involved in cell motility were excluded from this denomination. The authors argue that migrastatics should define a group of drugs that, by targeting essential end-point systems involved in motility, most likely avoid drug-resistance, as upstream signaling pathways are mostly redundant as well as involved in other cellular process [306].

A variety of CaM antagonists have been assayed for their ability to inhibit the enhanced migratory capacity of tumor cells, which is required for their invasiveness into adjacent tissues as well as blood and lymphatic dissemination of individual and, most efficiently, clusters of neoplastic cells forming metastasis (see Table 2).

Hypoxic conditions facilitate the attachment of human carcinoma HeLa cells to endothelial cells, and this is accompanied by the reorganization of the cytoskeleton and the redistribution of CaM to points of cell–cell contact. These processes are inhibited by the CaM antagonist W-7, preventing thereby the adhesion of the tumor cells to the endothelium [81]. The interaction of the migrated tumor cells with parenchymal cells of the invaded tissues is also of importance for the establishment of metastatic foci. The adhesion of mammary carcinoma and lymphosarcoma cells to rat hepatocytes has been studied in the context of liver metastasis. It was shown that the CaM inhibitor trifluoperazine inhibited the adhesion of the lymphosarcoma and breast carcinoma cells to the hepatocytes, albeit more efficiently in the former cells [79], suggesting the involvement of CaM in this process. Trifluoperazine also inhibited the migration and invasion capacity of a human colon carcinoma cell line as assayed in vitro. Most interestingly, this CaM antagonist also inhibited EMT in vivo as evidenced by the decreased expression of the EMT markers N-cadherin and the transcription factors Snail and Slug, increasing on the other hand the level of E-cadherin in the tumor cells [80].

The p68 RNA helicase is a Ca^2+^/CaM-binding protein that acts as a microtubule motor to transport Ca^2+^/CaM as a cargo to the leading edge of migrating cells. A peptide comprising the IQ motif of p68 inhibits cell migration and metastasis development by acting as a decoy preventing p68/CaM binding [307]. It could be of interest to determine whether similar processes occur during CaM distribution to the region of contact between tumor and normal cells, and during adhesion and invasiveness. PCP4/PEP19 (Purkinje cell protein 4/peptide 19) is a small protein with CaM-binding properties as described for IQSec/BRAG. It has anti-apoptotic properties [308] and is also implicated in cell adhesion, migration, and the invasiveness of breast tumor cells as inferred by knocking down its expression with siRNA [309]. However, how CaM regulates, if at all, the migratory function of this protein is not yet known and deserve investigation.

Calmodulin-regulated spectrin-associated proteins (CAMSAPs) are microtubule minus-end-targeting proteins involved in microtubule organization and dynamics by protecting the microtubules from kinesin-13-induced depolymerization, particularly CAMSAP2/3 but not CAMSAP1 [310] (reviewed in [311]). CAMSAP1 is overexpressed in laryngeal squamous cell carcinoma and its expression was shown to be inhibited by miRNA-126. Although no correlation between the plasma levels of miRNA-126 and lymph node metastasis was found, its inhibitory action on CAMSAP1 may explain the expected dysregulation of microtubule dynamics and why loss of miRNA-126 is frequently associated with tumor metastasis [312]. Furthermore, deletion of CAMSAP3 in human lung carcinoma-derived cells promotes EMT by a mechanism implicating tubulin acetylation and the subsequent activation of the Akt pathway [313]. The role of microtubules in cell migration encompasses additional mechanisms, including other CaM-dependent systems. CaMK-II was demonstrated to phosphorylate the microtubule destabilizer protein stathmin at Ser16, a process that is aided by Siva1 a protein that inhibits stathmin activity promoting α-tubulin/β-tubulin polymerization stabilizing the microtubules. Therefore, this protein blocks the assembly of focal adhesions, cell migration and EMT [314]. The authors of this study also demonstrated that highly metastatic breast carcinoma cell lines and human breast tumor biopsy samples present lower expression of Siva1 and decreased phospho-Ser16-stathmin levels, as compared to low metastatic breast tumor cell lines and normal breast tissues, consistent with the anti-metastatic role of the Siva1/CaMK-II system.

CaM inhibitors have been used not only to understand the functionality of many signaling pathways in normal and tumor cells, but also to inhibit critical tumor functions in the hope of using them as therapeutic agents. (reviewed in [27]). Although the latter is unlikely to be realized due to the universality of CaM regulating a great variety of cellular functions, as demonstrated by inducing CaM downregulation in an engineered conditional knock-out tumor cell system [315].

Lewis lung carcinoma is a syngeneic orthotopic mouse model of NSCLC widely used in experimental oncology (reviewed in [316]). In this model W-7 was tested for its anti-metastatic activity. Intravenous injection of this agent enhanced the number of peritoneal macrophages and inhibited lung metastasis of ectopically implanted tumor cells. The same effect was observed upon intravenous injection of W-7-activated macrophages [82]. The authors suggested that this effect may be due to activation of the C3 receptor of the complement system and macrophage activation. These results indicate that CaM in macrophages participates in the metastatic process. The results obtained in this murine model, however, do not necessarily lead to useful insights into the biology of human cancers. In any event, the CaM antagonist CBP501, a modified-peptide, inhibited human NSCLC cell migration, EGF-induced invasiveness, and EMT by blocking the interaction between CaM with K-Ras, as well as metastasis formation by Lewis lung carcinoma cells, leading to inhibition of cytokine production by the tumor-associated macrophages [71,72].

W-7 also downregulates the metastatic-associated suppressor genes *MTS1* (multiple tumor suppressor 1) and *NM23* in mouse melanoma [86]. These genes respectively code for the Ca^2+^-binding protein S100A4/metastasin 1 [317] and nucleoside diphosphate kinase-1 (reviewed in [318,319]). Interestingly, there are CaM-binding proteins that share the same binding site for Ca^2+^/S100 and Ca^2+^/CaM, for example, the ryanodine receptor [320,321] or peptides derived from the actin-capping protein TRTK12 and the tumor suppressor p53 [322].

The NDKase from the flagellar apparatus of the green algae *Chlamydomonas* contains three IQ motifs that allow Ca^2+^-dependent and Ca^2+^-independent CaM-binding [323]. It could be of interest to determine whether NDKase-1 is also a CaM-binding protein, which may explain at least in part the effect of W-7. 12-*O*-tetradecanoylphorbol-13-acetate (TPA)-induced expression of integrin-α_2_β_1_ in melanoma cells and the binding to its ligand, collagen type I, required for the invasiveness of the tumor cells, was also demonstrated to be inhibited by W-7 [87]. In addition, W-7 and down-regulating CaM with shRNA inhibited matrix metalloprotease activity required for the invasiveness of glioblastoma cells [237]. Phenoxybenzamine is a less known CaM antagonist that also exerts inhibitory action on α-adrenergic receptors [324]. This drug has been shown to inhibit the proliferation, migration, and invasiveness of different glioma cell lines, and it suppresses their tumorigenic potential by inhibiting the TrkB-Akt pathway [325]. Furthermore, two other CaM antagonists, J8 (*N*-8-aminooctyl-5-iodo-naphthalenesulfonamide) and tamoxifen, have the capacity to inhibit attachment of melanoma cells to the extracellular matrix and hence their invasiveness [74,78,326].

The anti-estrogenic action of tamoxifen may be explained at least in part by its antagonistic action on CaM, as the estrogen receptor binds and is activated by CaM [327] (reviewed in [328]). In this context, the pineal hormone melatonin has been described as having anti-metastatic properties by interfering with the interaction between CaM and the estrogen receptor preventing its activation (reviewed in [329]). Tamoxifen also blocks Ca^2+^ uptake in PC12 pheochromocytoma cells via voltage-dependent Ca^2+^ channels [330], which are regulated by CaM, and possibly by its antagonistic action on CaM, possibly contributing to its anti-metastatic action. Tamoxifen was also investigated in an experimental system of bone marrow metastasis by a neuroblastoma cell line, and it was observed that this agent inhibited the establishment of the tumor cells in a dose-dependent manner [77].

Metformin is an oral anti-diabetic type-II drug that has been associated with some tumor-protective function. This drug has been shown to inhibit the migration and invasiveness of a human fibrosarcoma cell line concomitant with the inhibition of phorbol-12-myristate-13-acetate (PMA)-induced Ca^2+^ influx and the secretion of MMP-9. Moreover, the intracellular Ca^2+^ chelator BAPTA-AM (1,2-bis(2-aminophenoxy)ethane-*N*,*N*,*N*′,*N*′-tetraacetic acid) and W-7 decreased both the PMA-induced secretion of this extracellular protease and tumor cell migration [83], suggesting a Ca^2+^-dependent action of CaM in these processes. 

## 6. Perspectives

A conditional tetracycline-dependent CaM-knockout system in chicken lymphoma DT40 cells has recently been developed [315]. In this system, the essential role of Ca^2+^/CaM for cell viability and for ligand-dependent activation of the EGFR during CaM downregulation was tested [315,331]. It could be interesting to develop similar conditional CaM-knockout systems in xenografted tumor cells to study the role of CaM in the development of metastasis in vivo. This could be done upon administrating the repressor, which downregulates CaM expression during different phases of the metastatic process, encompassing for example the local infiltration of normal tissues surrounding the primary tumor derived from implanted tumor cells, the transmigration of these cells through blood and lymphatic vessels, the invasion of local lymph nodes, the viability of the circulating tumor cells, and/or the establishment of metastatic foci in colonized distant organs. This would allow determination of the step(s) where CaM is unequivocally essential and most likely to be targeted with different inhibitors to prevent metastasis.

Another interesting line to follow relevant to understanding the signaling mechanisms responsible for steering the cell toward adequate paths involving Ca^2+^ flickering events in the advancing front edge of the cell moving toward/avoiding chemo/mechanical gradients or cues [3], reviewed in [4] (see Figure 1), would be to determine the exact role of Ca^2+^/CaM in this process, and most significantly, to identify the essential CaM-dependent systems intervening in the underlying signaling pathways. In this context, it is important to consider that the primary cilium is a small non-motile centriole-associated microtubule-containing structure protruding from the plasma membrane of interphase cells with essential signaling functions via the Hedgehog pathways in vertebrate cells. This organelle has been considered a cell antenna with chemical, temperature, and mechanotransduction properties, and is implicated among other functions in cell migration (reviewed in [332,333,334]). Mutations of ciliary proteins are responsible for the occurrence of important congenital defects denoted non-motile ciliopathies (reviewed in [335]). The primary cilium participates in Ca^2+^ signaling via a variety of TRP channels (reviewed in [336]), and direct visualization of Ca^2+^ mobilization within the primary cilium has been measured by a targeted genetically encoded Ca^2+^ sensor upon chemical and mechanical stimulation [337]. However, using comparable techniques, other authors concluded that no Ca^2+^ mobilization in the primary cilium was detected when cells were subjected to mechanical stimulation elicited by fluid flow [338], although the same group earlier demonstrated that the primary cilium has Ca^2+^ channels that mediate signaling functions [339,340]. Beside the fact that TRP channels are regulated by CaM [341], and that inversin/nephrocystin-2 and nephrocystin-5—conserved proteins in the proximal segment of the primary cilium—bind CaM [336,341], little is known about how CaM transduces Ca^2+^ events in this organelle, and what role it would play during cell migration dependent on primary cilia. Moreover, whether CaM mutations could affect the functionality of the primary cilium by interfering with CaM-dependent ion channels as shown in other systems [342], and hence the migratory capacity of the cells, is an interesting open question that deserves future investigation.

CaM antagonists have been tested as therapeutic agents in cultured tumor cells, and in restricted clinical assays as co-adjuvant in some patients in combination with anti-tumor drugs used in chemotherapy to inhibit CaM-dependent systems in the tumor cells to prevent cell growth, invasiveness and/or metastasis development yielding some positive results (reviewed in [27] and Table 2). However, these drugs are unlikely to be useful for a wide application in the clinic due to the expected adverse effects caused by the pleiotropic action of CaM controlling multiple vital functions in all normal cells. An alternative option, however, could be to selectively target specific hyperactive or overexpressed CaM-dependent proteins in tumor cells by blocking their respective CaM-binding sites as we have previously suggested (reviewed in [27,343]).

## Figures and Tables

**Table 1:** Selected examples of CaM antagonists inhibiting non-tumor cell migration.

CaM Antagonist	Cell Type (Species)	Effects	Mechanisms	Ref.
Calmidazolium (R24571)	Cerebellar granule cells (mice) Gastric epithelial cells (rabbit)	Inhibits cell migration	Reduces Ca^2+^ transients	[50,61]
Chlorpromazine	WIRL liver epithelial cells (rat)	Inhibits cell spreading and decreases F-actin levels	Alters microfilaments function; inhibits actin polymerization	[45]
Fluphenazine	Fibroblasts (human) ^(1)^ Fibroblasts (rat)	Inhibits cell migration and contractility; inhibits wound contraction and re-epithelization in vivo	Inhibits MLCK	[62,63]
Trifluoperazine	3T3 fibroblasts (mouse) SV40 transformed 3T3 fibroblasts (mouse) WIRL liver epithelial cells (rat) SV40 transformed WIRL cells (rat) NRK-LA23 kidney cells (rat) NIL-2 fibroblasts (hamster) Fibroblasts (human) MRC-5 diploid fibroblasts (human) Aortic smooth muscle cells (rat) Neutrophils (human) ^(2)^ ECV304 umbilical vein endothelial cells (human) Corneal epithelial cells (rat) Monocytes (human) ^(3)^	Inhibits cell migration, cell spreading, cell adhesion; decreases F-actin levels; inhibits ATP-induced cell contraction and prevents MLC phosphorylation	Alters microfilaments function;inhibits actin polymerization and aggregation; inhibits MLCK and actomyosin function	[45,46,48,55,64,65]
W-7	CFSC-8B myofibroblast-like hepatic stellate cells (rat) Bronchial epithelial cells (human) ECV304 umbilical vein endothelial cells (human) Corneal epithelial cells (rat) Gastric mucosal cells (rabbit) Neutrophils (human) Myofibroblasts (rat) Vascular smooth muscle cells (rat) Fibroblasts (rat)	Inhibits PDGF-mediated cell migration; inhibits VIP-, IL-8-, CT-1-induced chemotaxis; inhibits wound contraction and re-epithelization in vivo	Inhibits CaMK-II, MLCK and actomyosin function	[49,51,55,63,64,66,67,68,69]

^(1)^ Isolated from normal donors and Dupuytren’s disease patients. ^(2)^ Inhibits MLCK in the endothelium preventing neutrophils transmigration. ^(3)^ Very small inhibitory effect. CaMK-II, calmodulin-dependent protein kinase-II; CT-1, cardiotrophin-1; IL-8, interleukin-8; MLC, myosin light-chain; MLCK, myosin light-chain kinase; SV40, simian virus 40; VIP, vasoactive intestinal peptide.

**Table 2:** Selected examples of CaM antagonists inhibiting tumor cell migration and invasiveness.

CaM Antagonist	Cell Type (Species)	Effects	Mechanisms	Ref.
Calmidazolium (R24571)	Colo201 colon cancer cells (human)	Inhibits cell adhesion	Inhibits calcineurin	[70]
CBP501	A549 NSLC cells (human) H1299 NSLC cells (human) Lewis lung carcinoma cells (mouse) ^(1)^	Inhibits EGF-dependent cell migration and invasiveness; inhibits EMT; inhibits metastasis; inhibits tumor spheroid formation	Inhibits CaM binding to K-Ras; inhibits Akt and ERK1/2 activation; inhibits EGF-induced transcription factor Zeb1 and vimentin expression; inhibits tumor-promoting IL-6 and TNFα production by tumor-promoting macrophages	[71,72]
Chlorpromazine	MOLT-4 lymphoma T cells (human)	Inhibits invasiveness	Inhibits actin polymerization; inhibits CaMK-II	[47,73]
Diphenylbutylpiperidine	MOLT-4 lymphoma T cells (human)	Inhibits invasiveness	Inhibits CaMK-II	[73]
Flunarizine	B16 melanoma cells (mouse) K1735-M2 melanoma cells (mouse) M5076 macrophage-like sarcoma cells (mouse)	Inhibits cell migration and cell invasiveness		[53,54]
J8	A375-SM melanoma cells (human) Choroidal melanoma cells (human)	Inhibits cell invasiveness		[74,75]
Ophiobolin A	HeLa cervix carcinoma cells (human) PC3 prostate carcinoma cells (human)	Inhibits cell migration and enhances Ca^2+^ leak from ER	Prevents CaM binding to Sec61 Ca^2+^ leak channel and increases SOCE	[59]
Prochlorperazine	MOLT-4 lymphoma T cells (human)	Decreases F-actin	Inhibits actin polymerization	[47]
Squalamine	13762 mammary carcinoma (rat) Lewis lung carcinoma (mouse) MV-522 lung carcinoma (human) Calu-6 lung adenocarcinoma (human) H460 large-cell lung carcinoma (human) NCI-H23 non-small cell lung carcinoma (human)	Synergistic anti-metastatic action in combination with different anti-tumor agents	Disrupts F-actin stress fibers	[76]
Tamoxifen	C-1300 neuroblastoma cells (mice) A375-SM melanoma cells (human) Uveal melanoma cells (human) Choroidal melanoma cells (human) B16 melanoma cells (mouse) MCF7 breast carcinoma cells (human)	Inhibits cell attachment; inhibits invasiveness; inhibits bone marrow metastasis		[74,75,77,78]
Trifluoperazine	HeLa cervix carcinoma cells (human) PC3 prostate carcinoma cells (human) TA3 breast carcinoma cells (mouse) MB6A lymphosarcoma cells (mouse) HCT116 colon carcinoma cells (human)	Inhibits cell migration, invasiveness, EMT and enhances Ca^2+^ leak from ER	Prevents CaM binding to Sec61 Ca^2+^ leak channel; increases SOCE; inhibits adhesion to hepatocytes and decreases expression of N-cadherin and the transcription factors Snail and Slug; inhibits CaMK-II	[59,79,80]
Triflupromazine	MOLT-4 lymphoma T cells (human)	Decreases F-actin	Inhibits actin polymerization	[47]
W-7	HeLa cervix carcinoma cells (human) Lewis lung carcinoma cells (mouse) HT-1080 fibrosarcoma cells (human) MOLT-4 lymphoma T cells (human) CHRF-28-11 megakaryoblastic leukemia cells (human) Colo201 colon cancer cells (human) B16 melanoma cells (mouse) SK-MEL118 melanoma cells (human)	Inhibits cell migration, cell adhesion, cell invasiveness, and metastasis in vivo	Decreases PMA-induced MMP-9 secretion; inhibits EGF- and thrombin-dependent Rac1 activation; inhibits calcineurin; inhibits TPA-induced integrin-α_2_/β_1_ expression; inhibits *MTS1* and *NM23* metastasis-associated genes	[70,73,81,82,83,84,85,86,87]

^(1)^ Indirect action blocking production of cytokines by tumor-promoting macrophages in co-culture. EGF, epidermal growth factor; EMT, epithelial-mesenchymal transition; ER, endoplasmic reticulum; IL-6, interleukin-6; MMP-9, matrix metalloprotease-9; NSLC, non-small lung carcinoma; PMA, phorbol-12-myristate-13-acetate; SOCE, store-operated Ca^2+^ entry; TNFα, tumor necrosis factor-α; TPA, 12-*O*-tetradecanoylphorbol-13-acetate; Zeb1, zinc finger E-box-binding homeobox 1.
